# Microbiological and Chemical Profiles of Kiwi Kefir-like Beverages Produced Using Different Agitation Speeds and Kefir Grain Weights

**DOI:** 10.3390/foods14101681

**Published:** 2025-05-09

**Authors:** Delicia L. Bazán, Pablo G. Del-Río, Nelson Pérez-Guerra

**Affiliations:** 1 Departamento Académico de Ingeniería de Industrias Alimentarias, Facultad de Ingeniería, Universidad Nacional de Jaén, Carretera Jaén-San Ignacio KM 24-Sect., Yanuyacu, Jaén 06801, Peru; delicia.bazan@unj.edu.pe; 2 Departamento de Química Analítica e Alimentaria, Facultade de Ciencias, Universidade de Vigo, 32004 Ourense, Spain; 3 Departamento de Enxeñaría Química, Facultade de Ciencias, Universidade de Vigo, 32004 Ourense, Spain; pdelrio@uvigo.es; 4 Instituto de Agroecoloxía e Alimentación (IAA), Campus Auga, Universidade de Vigo, 32004 Ourense, Spain

**Keywords:** kiwi kefir-like beverage, kefir grain weight, agitation speed, lactic acid bacteria, yeasts, batch fermentation

## Abstract

This study aimed to identify kiwi kefir-like beverages with high levels of viable probiotic cells and low levels of calories, acids, and alcohol. To achieve this, microbiological and chemical characterizations were conducted on beverages inoculated with varying amounts of kefir grains (GW) and incubated at different agitation speeds (A), following a second-order orthogonal factorial design. For each experimental condition, three 24-h batch cultures were performed using three successive passages of kefir grains. Higher GW levels promoted greater nutrient consumption and metabolite production. However, an intermediate GW (1.80 g) resulted in the highest growth of lactic acid bacteria (LAB), acetic acid bacteria (AAB), yeasts, and free biomass in the fermented medium. Optimal agitation levels also enhanced nutrient consumption, free biomass, and metabolite pro-duction. AAB and yeast counts increased with higher agitation speeds, while LAB counts de-creased. Three beverages, produced during the second (A = 86 rpm, GW = 2.81 g) and third (A = 38 rpm, GW = 2.60 g; A = 86 rpm, GW = 1.80 g) kefir grain passages, exhibited LAB and yeast counts above 10^6^; CFU/mL, along with low total sugar and ethanol concentrations. These beverages may be considered suitable as potentially probiotic, low-alcohol, and low-calorie functional drinks.

## 1. Introduction

Kiwifruit is enjoyed by people worldwide for its appealing taste, nutritional benefits, health advantages, and acidic flavor and the refreshing aroma of its pulp, factors that contribute to the popularity of this fruit among consumers [1]. Although the most commercially grown kiwifruit is *Actinidia deliciosa* (known as green kiwi), *A. chinensis* (gold kiwi) and *A. argute* (baby kiwi) are also being produced worldwide [2].

This fruit is a high-value crop that has gained significant acceptance both nationally and internationally due to its high levels of vitamins C, E, and B (particularly folic acid, or vitamin B9) [3]. It also contains minerals such as potassium, calcium, and phosphorus, as well as dietary fiber and polyphenols [4].

Consuming this fruit offers digestive advantages as it aids in stomach and ileum digestion, helping to relieve constipation and symptoms of irritable bowel syndrome [5]. It also possesses properties that help combat diabetes, tumors, inflammation, ulcers, and oxidative stress while supporting the regulation of blood sugar and lipid levels. Additionally, it has shown benefits in managing conditions such as edema, hepatitis, kidney disorders, rheumatoid arthritis, and microbial infections [6].

In Spain, the final production estimates for fruit crops for the 2024–2025 harvest predict an 18.0% increase for kiwi (31.7 million tons) compared to the previous season and a 17.4% increase compared to the average of the past five seasons (2019–2023) [7].

However, many kiwifruits have low commercial value due to their small size [2], resulting in these fruits being discarded. This leads to a significant impact on sales and, consequently, on the incomes of growers in the Spanish regions of Galicia and Asturias, which are the main kiwifruit-producing areas. Therefore, it is necessary to design alternative methods to add value to small-caliber kiwifruits.

Some researchers have explored the production of non-dairy kefir-like beverages through the fermentation of fruit juices such as apple, quince, grape, kiwifruit, prickly pear, and pomegranate [8]; orange, apple, dragon fruit, and kiwifruit [9]; red table grapes [10]; and chestnut puree [11]. Given the promising results of these studies, fermenting kiwi juice with kefir grains to produce a kefir-like beverage could provide a valuable alternative to mitigate losses in the kiwifruit harvest. This approach would not only preserve the health benefits of kiwi juice but also enhance them with the positive effects of the microorganisms present in the kefir grains [8,10,12].

Typically, the production of kefir beverages has been carried out under static conditions [13,14,15,16,17]. However, numerous studies have shown that agitation can have varying effects on both the growth of kefir grains and the production of fermentation metabolites [18,19,20,21,22,23]. An agitation speed of 130 rpm increases kefir grain mass, the production of organic acids, and a reduction in pH values of the culture medium in pasteurized full-cream or low-fat milk compared to the culture performed without agitation [18]. Güzel-Seydim et al. [19] also indicated that agitation speeds between 75 and 100 rpm are optimal for producing kefir from the fermentation of pasteurized milk with kefir grains. Additionally, increasing the agitation speed from 0 to 80 rpm enhanced kefir grain growth and kefiran production, likely due to improved mass transfer and more uniform temperature distribution within the fermenter [20]. However, further increasing the speed from 80 to 160 rpm negatively affected both kefir grain growth and kefiran production, probably due to excessive fragmentation of the grains [20]. In contrast, an agitation speed of 125 rpm was identified as optimal for maximum kefir grain biomass production, with growth decreasing at both lower and higher speeds [21].

In another study, an increase in agitation speed from 50 to 250 rpm led to a reduction in kefir grain growth and a decrease in the production of lactic acid and kefiran in skimmed milk compared to the culture incubated under static conditions [22]. Similarly, an increase in agitation speed from 60 to 90 rpm resulted in a dramatic decrease in kefir grain growth, although this change did not affect kefiran production in whey [23].

Regarding the inoculum, microorganisms isolated from water kefir were used to ferment Mediterranean fruit [8] and vegetable [16] juices, while those isolated from milk kefir grains were used to inoculate cow’s milk [24]. Additionally, different kefir beverages were produced by fermenting red table grape juice [10], chestnut puree [11], chokeberry juice [12], and dairy food processing by-products (such as acid whey permeate, buttermilk, sweet whey permeate, and sweet whey permeate with added milk fat globules) [17] with milk kefir grains. Similarly, water kefir grains were used as inoculum to ferment apple, orange, dragon fruit, and kiwifruit juices [9]; a water kefir simulation medium (prepared with fig extract, unrefined cane sugar, and distilled water) [25]; or a solution remaining from the osmotic dehydration of pineapple [26].

Although different agitation speeds (A) and kefir grain weights (GW) have been used in the production of kefir beverages [13,14,15,16,17,18,19,20,21,22,23], no studies have evaluated the combined effect of these two variables on kefir production. Therefore, selecting the optimal levels of A and GW for kefir beverage production remains challenging.

This study aims to identify potential functional kiwifruit kefir-like beverages with high counts of viable probiotic cells and low caloric, alcoholic, and acidic contents, produced by inoculating the beverages with varying kefir grain weights and incubating them at different agitation speeds. In the present study, milk kefir grains were used instead of water kefir grains to ferment kiwi juice, aiming to expand the range of products that can be produced with this type of grains. To properly organize the experiments, the beverages were produced through 24 h batch fermentations over three successive kefir grain passages (0–24 h, 24–48 h, and 48–72 h), following a second-order orthogonal factorial design.

The response variables in the experimental design included the consumption of total sugars and organic acids (citric and quinic acids); viable cell counts of lactic acid bacteria, acetic acid bacteria, and yeasts; and the production of free biomass, organic acids, alcohols, and antibacterial activity in each subculture. This design also facilitated the determination of the optimal agitation speed and kefir grain weight for each dependent variable.

Additionally, principal component analysis (PCA) was applied to identify similarities and differences among the beverages based on their microbiological and chemical profiles, thereby enabling the selection of the most suitable functional beverages.

## 2. Materials and Methods

### 2.1. Preparation of Kefir Grains and Kiwi Juice

The milk kefir grains embedded in milk (cover liquid) were purchased from Kefiralia (Burumart Commerce S.L., Arrasate, Guipúzcoa, Spain). The activation of the kefir grains was carried out in fresh ultra-high-temperature (UHT) whole milk (Central Lechera Asturiana, Asturias, Spain) following the method described by Bazán et al. [27]. The counts of LAB, AAB, and yeasts in these kefir grains were 9.9 ± 1.3 × 10^7^, 6.8 ± 1.0 × 10^6^, and 7.8 ± 1.6 × 10^7^ CFU/g, respectively [10].

Kiwifruit (*Actinidia deliciosa* var. Hayward) was sourced from a local plantation (Noia, La Coruña, Spain). The fruits were chosen due to their common availability in Spanish supermarkets, making them easily accessible to consumers who wish to ferment kiwi juice with kefir grains in an artisanal manner.

To obtain kiwi juice, the fruits were peeled and blended in a mixer, ensuring that the seeds were not crushed. The resulting pulp was then filtered using a sterile plastic strainer, and the filtered volume was measured. To calculate the juice yield, the peels, peeled fruit, remaining solids from the pulp filtration, and the filtered liquid were weighed.

To remove suspended solids, the filtered kiwi juice was centrifuged (Avanti J–26 XP centrifuge, Beckman Coulter, Fullerton, CA, USA) at 15,000 rpm for 15 min at 4 °C, and the precipitate was discarded, but the supernatant (centrifuged kiwi juice) was reserved for use as a substrate in kefir grain fermentations. The yield obtained from kiwi juice extraction was 288 mL of filtered kiwi juice per kg of fruit. The initial mean composition (g/L) of the kiwi juice used as the fermentation substrate was as follows: total sugars, 123.56 ± 0.05; citric acid, 17.27 ± 1.06; quinic acid, 9.30 ± 0.06; malic acid, 0.17 ± 0.01; proteins, 2.09 ± 0.04; total nitrogen, 0.33 ± 0.01; and pH 3.27 ± 0.17. The counts of lactic acid bacteria (LAB), Enterobacteriaceae, *Pseudomonas*, acetic acid bacteria (AAB), and yeasts in the centrifuged juice were below the minimum detection limit of 30–300 colonies.

### 2.2. Fermentation Conditions

Fermentation was conducted in duplicate at room temperature in 150 mL glass jars which were previously sterilized, containing 60 mL of kiwi juice without supplementation. Each medium was inoculated with the corresponding grain weight, and the mouth of each jar was covered with a sterile, breathable cloth secured by a rubber band. The jars were then incubated at the corresponding agitation speed at room temperature for 24 h (first subculture), avoiding exposure to direct sunlight during incubation [27].

**Figure 1:**
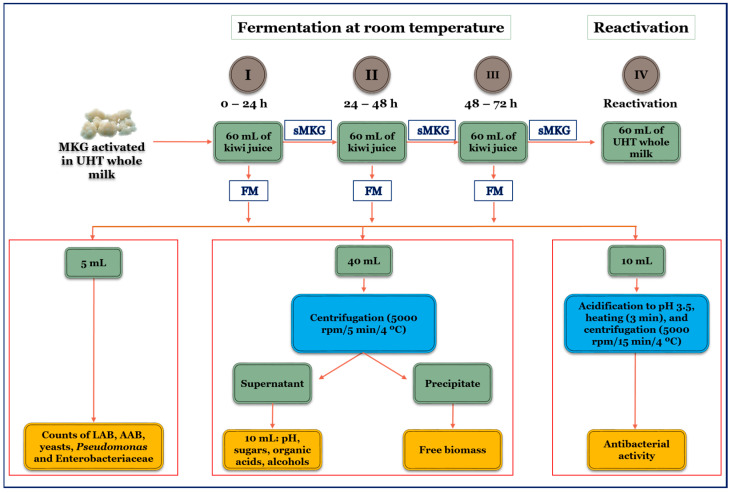
A block diagram of the experimental procedure used for the production and analysis of the kiwi kefir-like beverages during the first (0–24 h), second (24–48 h), and third (48–72 h) subcultures in kiwi juice. FM: filtered fermented medium; sMKG: milk kefir grains separated from the fermented medium by filtration.

The three consecutive subcultures of 24 h were performed as indicated in Figure 1 [10,27]. After each subculture, the kefir grains were separated from the fermented medium using a sterile plastic strainer, washed with mineral water (Aguas de Cabreiroá, S.A.U., Ourense, Spain), and air-dried for 10 min on tissue paper under aseptic conditions in a biosafety cabinet. The grains were then used to inoculate 60 mL of fresh kiwi juice for the second or third subculture [10].

The filtered fermented medium (Figure 1) was divided into three aliquots for characterization.

The first aliquot (5 mL) was employed to quantify the colony-forming units (CFUs) of lactic acid bacteria (LAB), acetic acid bacteria (AAB), yeasts, *Pseudomonas*, and Enterobacteriaceae present in the fermented beverages. The second aliquot (40 mL) was centrifuged (5000 rpm, 5 min, 4 °C), and the supernatant was used to measure the culture pH and the concentrations of sugars, organic acids, and alcohols. The precipitate was used to quantify the free biomass released from the grains into the fermented kiwi juice. The third aliquot (10 mL) was used to assess the antibacterial activity in the fermented samples.

After the third fermentation process in kiwi juice (a non-dairy substrate), the milk kefir grains were reactivated by introducing them into UHT whole milk (Central Lechera Asturiana, Asturias, Spain) for six 24 h incubation periods under static conditions. Following the final passage, the milk kefir grains, submerged in milk, were stored at 4 °C until further use.

### 2.3. Microbiological and Chemical Analysis of Unfermented Kiwi Juice and Kiwi Kefir-like Beverages

The enumeration of LAB, AAB, yeasts, *Pseudomonas*, and Enterobacteriaceae in the centrifuged kiwi fruit juice and fermented beverages was performed in triplicate as described in previous studies [8,10,16]. Serial dilutions of the samples were plated on de Man, Rogosa, and Sharpe agar (MRSA); Carr agar (CA); yeast extract–glucose agar (YEGA); *Pseudomonas* agar base supplemented with 10 g/L cetrimide and fucidin (PAB-CF); and double-layered violet red bile glucose agar (VRBGA). After sterilization, MRS and CA media were supplemented with 0.1 g/L amphotericin B (resulting in MRSA-A and CA-A media, respectively) to prevent fungal growth, while YEGA was supplemented with 0.1 g/L chloramphenicol (YEGA-C medium) to inhibit bacterial growth. The MRSA-A, CA-A, YEGA-C, PAB-CF, and VRBGA plates were incubated under the following conditions: 30 °C for 48 h (anaerobically), 30 °C for 48 h, 25 °C for 48 h, 20 °C for 48 h, and 37 °C for 24 h, respectively [8,10,16]. After incubation, the enumeration of LAB, AAB, yeasts, *Pseudomonas*, and Enterobacteriaceae was carried out using the MRSA-A, CA-A, YEGA-C, PAB-CF, and VRBGA plates, respectively.

The concentrations (in g/L) of total sugars (TSs, defined as the sum of glucose and fructose), organic acids, and alcohols in the centrifuged kiwi fruit juice and fermented beverages were quantified in triplicate by high-performance liquid chromatography [10]. Standards consisted of solutions of glucose, fructose, citric acid, quinic acid, lactic acid, acetic acid, ethanol, and glycerol, all at 10 g/L. Both standards and centrifuged samples of kiwi fruit juice and fermented beverages were filtered and, if necessary, diluted with distilled water. An aqueous solution of 0.012 N sulfuric acid was used as the mobile phase, with a flow rate of 0.4 mL/min, to separate the chemical compounds [10].

To quantify the free biomass released from the grains into the fermented kiwi juice, the precipitates obtained after centrifuging the second aliquot were washed twice with saline (0.8% *w*/*v* NaCl). Following centrifugation (5000 rpm, 5 min, 4 °C), the pellets were resuspended in saline, and the absorbance of these suspensions at 700 nm was converted to dry cell weight using a previously established standard curve [10].

The concentrations of proteins and nitrogen in the unfermented kiwi juice were determined in triplicate using the method of Lowry et al. [28], with bovine serum albumin as the standard, and the micro-Kjeldahl method [29], with ammonium sulfate as the standard, respectively.

### 2.4. Antibacterial Activity of Kiwi Kefir-like Beverages

The antibacterial activity of unfermented kiwifruit juice and kefir-like kiwi beverages was assessed in triplicate, as described by Costas et al. [30], using *Carnobacterium piscicola* CECT 4020 as the indicator strain. The samples were acidified to pH 3.5 to prevent the adsorption of bacteriocins onto the cell wall of bacteriocin-producing strains. They were then heated for 3 min in a boiling water bath to kill the cells, which were subsequently separated from the supernatant by centrifugation (5000 rpm, 15 min at 4 °C). The supernatants, which contained antibacterial activity (comprising bacteriocins, organic acids, and alcohols), were adjusted to pH 6.0 and appropriately diluted with sterile distilled water. Equal volumes of diluted supernatants and a culture of *C. piscicola* CECT 4020 in buffered MRS broth (adjusted to an initial absorbance of 0.2 at 700 nm) were then mixed in culture tubes and incubated at 30 °C for 6 h. Control samples (in triplicate) consisted of three culture tubes containing equal volumes of sterile 0.1 M sodium biphthalate buffer (pH 6.3) and the *C. piscicola* CECT 4020 culture.

Growth inhibition (GI) was determined by measuring the absorbance of the incubated samples at 700 nm and comparing it to the absorbance of the controls, calculated as follows:

$$GI=1-\frac{Am}{Ac}$$

where Am and Ac are the absorbances of the sample and the control, respectively.

The antibacterial activity (in activity units (AUs) per mL) was expressed as the inverse of the dilution that caused 50% growth inhibition (the inhibitory dose was 50), which was determined from dose–response curves [30].

### 2.5. Statistical Methods

#### 2.5.1. Experimental Design

The effect of agitation speed (A) and kefir grain weight (GW) on various culture variables (such as total sugar and organic acid consumption, growth, and product synthesis) during the fermentation of kiwi juice with milk kefir grains was investigated using a second-order orthogonal factorial design. To achieve this, batch cultures were developed and divided into three consecutive 24 h subcultures (0–24 h, 24–48 h, and 48–72 h) at each point of the experimental design.

Table 1 shows the experimental domains and the natural and coded values of the independent variables. The experimental domain for variable A was selected by using an agitation range of 25 to 150 rpm (the latter was used by our research group in previous experiments). Although it would seem logical to use the level of rpm = 0 (no agitation, corresponding to the coded value of −1.267) as the lower limit in the experimental design, such an approach became unfeasible as its use would imply that the coded value of −1 would correspond to a natural value of 16 rpm, which could not be achieved with the agitator used (Innova 4330, New Brunswick Scientific, Edison, NJ, USA). For this reason, the minimum level (−1.267) for variable A was selected as the lowest agitation speed (25 rpm) that allowed the equipment to be used.

The experimental domain for variable GW was selected by taking as the lower limit a grain weight of 0.79 g in a 60 mL substrate volume (ratio: 13.17 g of kefir grain per L of fermentation medium) and a maximum level of 2.81 g in a 60 mL substrate volume (ratio: 46.83 g of kefir grain per L of fermentation medium), which includes, as the mean value, the inoculation ratio (30.80 g in 1 L of substrate) recommended by the grain supplier (Kefiralia, Burumart Commerce S.L.).

The statistical significance of the empirical models and their coefficients was analyzed using Fisher’s *F* test (*p* < 0.05) for analysis of variance (ANOVA) and Student’s *t*–test, with a significance level of α ≤ 0.05, respectively. Statistical analyses of all empirical models (see Tables S1–S36, Supplementary Material) were conducted using the experimental design module of the Statistica software package (Statistica 14.1.0 for Windows; StatSoft Inc., 2023, Tulsa, OK, USA). The different empirical models obtained contain only the parameters with statistically significant coefficient values (*p* < 0.05). 

#### 2.5.2. Principal Component Analysis

The relationship between the mean microbiological and chemical compositions in the 27 fermented beverages was studied through principal component analysis (PCA) using the Factor Analysis algorithm from the IBM^®^ SPSS^®^ Statistics 23 software package for Windows (version 23.0, Armonk, NY, USA: IBM Corp., 2015). Bartlett’s sphericity test was used to assess whether the principal component analysis was suitable for the experimental data.

This method reduces the original data matrix into one of the smaller dimensions, which accounts for most of the observed variability in the initial experimental data. Factors with eigenvalues greater than 1.0, as per the Kaiser criterion, were used for the principal component analysis [8].

The discrimination variables used were the counts (as log CFU/mL) of LAB, AAB, and yeasts, along with the concentrations (g/L) of free biomass (X) in the fermented medium, total sugars (TS), citric acid (CA), quinic acid (QA), lactic acid (LA), acetic acid (AA), ethanol (EtOH), and glycerol (GOH). The kiwi kefir-like beverages obtained during the first kefir grain passage (0–24 h) were labeled as Bev1-24 h, Bev2-24 h, Bev3-24 h, Bev4-24 h, Bev5-24 h, Bev6-24 h, Bev7-24 h, Bev8-24 h, and Bev9-24 h. The beverages obtained in the second kefir grain passage (24–48 h) were labeled as Bev1-48 h, Bev2-48 h, Bev3-48 h, Bev4-48 h, Bev5-48 h, Bev6-48 h, Bev7-48 h, Bev8-48 h, and Bev9-48 h. The beverages obtained in the third kefir grain passage (48–72 h) were labeled as Bev1-72 h, Bev2-72 h, Bev3-72 h, Bev4-72 h, Bev5-72 h, Bev6-72 h, Bev7-72 h, Bev8-72 h, and Bev9-72 h.

The microbial counts (LAB, AAB, and yeasts) and the concentrations of free biomass, total sugars, organic acids, and alcohols for the five beverages obtained from the center of the domain (A = 86 rpm, GW = 1.80 g) were averaged and used in the PCA. These beverages, collected during the first, second, and third kefir grain passages, were labeled as Bev9-24 h, Bev9-48 h, and Bev9-72 h, respectively.

## 3. Results and Discussion

### 3.1. Effect of Agitation and Kefir Grain Weight on Kinetics of Kiwi Juice Fermentation over Three Successive Kefir Grain Passages (0–24 h, 24–48 h, and 48–72 h)

In this study, kiwi kefir-like beverages were produced by inoculating kiwi juice with different kefir grain weights (GW) and incubating them at varying agitation speeds (A) over three consecutive kefir grain passages (0–24 h, 24–48 h, and 48–72 h).

Although the aim of this study was to identify kiwifruit beverages with high probiotic cell counts and low levels of calories, acids, and alcohol, the methodological approach employed also enabled the determination of the effects of the two independent variables (GW and A) on key culture parameters. These included the consumption of total sugars, citric acid, and quinic acid; the counts of LAB, AAB, and yeasts; and the production of free biomass, lactic acid, acetic acid, ethanol, glycerol, and antibacterial activity, as described below.

#### 3.1.1. Nutrient Consumption

The results show that the combined effect of agitation (A) and kefir grain weight (GW) on the consumption of total sugars ([TS]c, in g/L) across the different subcultures can be described by the following consistently adjustable second-order empirical equations (statistical analysis in Tables S1–S3):

[TS]c (24 h) = 51.99 + 10.87·A + 8.59·GW − 9.28·A^2^ − 9.11·GW^2^ (1)

[TS]c (48 h) = 57.51 + 12.52·A + 8.35·GW − 7.66·A^2^ (2)

[TS]c (72 h) = 77.88 + 9.52·A + 9.85·GW − 7.37·A^2^ (3)

**Figure 2:**
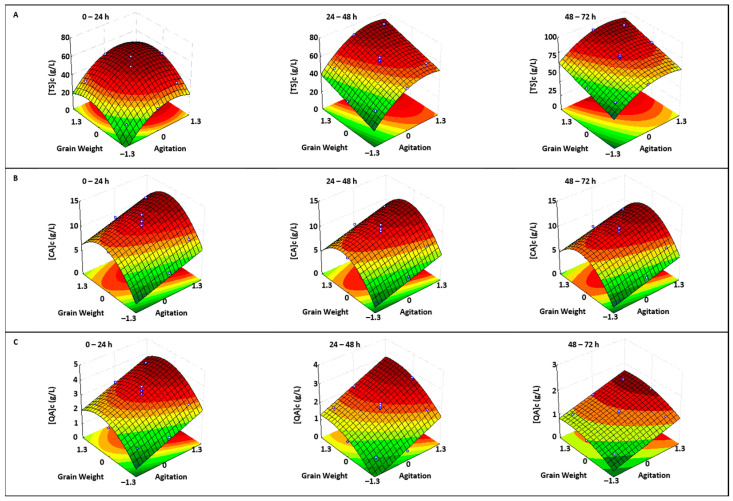
Response surfaces showing the effect of agitation and kefir grain weight on the consumption of (**A**): total sugars ([TS]c), (**B**): citric acid (CA), and (**C**): quinic acid (QA) in the first (0–24 h), second (24–48 h), and third (48–72 h) subcultures. The symbols (white circles) above and below the response surfaces represent the experimental data points.

As observed in the response surfaces (Figure 2A) generated from these empirical models (1–3), maximum total sugar (TS) consumption occurred at agitation speeds between 124 and 130 rpm and for kefir grain weights (GW) ≥ 2.18 g (see Tables S1–S3 in the Supplementary Materials). These results suggest that, with a higher inoculum level, increasing the agitation speed to its optimal value enhanced mass transfer phenomena, including (i) oxygen dissolution in the fermentation medium (important for AAB and yeasts), (ii) nutrient and O_2_ uptake by cells, and (iii) the release of gases and metabolic products from the cells into the fermentation medium [31,32]. However, when agitation exceeds its optimal value, shear forces can damage the bacterial and yeast-like cells present in the kefir grains [33,34].

As observed in Figure 2A, the maximum levels of total sugars consumed (57.20, 61.08, and 93.44 g/L) increased with the number of kefir grain passages, likely due to better adaptation of the kefir grain microorganisms to the acidic conditions of the fermentation substrate.

Regarding citric acid (CA) and quinic acid (QA), the results show a reduction in the concentrations of both acids at the end of the different subcultures, suggesting their assimilation by the kefir grain microorganisms. Although the production of organic acids (e.g., citric, lactic, and acetic acid) by yeasts, LAB, and AAB is a common feature of fermentations [35,36], it has also been observed that these microorganisms are capable of assimilating various organic acids (e.g., tartaric, acetic, lactic, malic, citric, and succinic acids) [37,38,39].

The assimilation of citric acid ([CA]c, in g/L) and quinic acid ([QA]c, in g/L) followed a similar pattern to that of total sugars (Figure 2A–C), as indicated by empirical models 4–9 (statistical analysis in Tables S4–S9):

[CA]c (24 h) = 11.37 + 1.75·A + 1.65·GW − 2.75·GW^2^ (4)

[CA]c (48 h) = 10.19 + 1.61·A + 1.38·GW − 2.78· GW^2^ (5)

[CA]c (72 h) = 9.65 + 1.43·A + 1.30·GW − 2.69· GW^2^ (6)

[QA]c (24 h) = 3.37 + 0.70·A + 0.54·GW − 0.64·GW^2^ (7)

[QA]c (48 h) = 1.91 + 0.66·A + 0.57·GW − 0.29· GW^2^ (8)

[QA]c (72 h) = 1.20 + 0.32·A + 0.22·GW − 0.14· GW^2^ (9)

The maximum citric acid consumption (CAmax = 13.83, 12.40, and 11.62 g/L) in the successive cultures (Tables S4–S6) occurred at the same agitation speed (147 rpm) and similar kefir grain weights (2.04, 2.00, and 1.99 g). Additionally, the maximum levels of assimilated quinic acid (QAmax = 4.37, 3.02, and 1.70 g/L) were consistently lower than those of citric acid at an agitation speed of 147 rpm and kefir grain weights of 2.13, 2.57, and 2.47 g (Tables S7–S9).

These decreasing levels of CAmax and QAmax contrast with the increasing optimal levels of total sugar consumption, suggesting that, as the number of subcultures increased, the kefir grain microorganisms preferentially utilized monosaccharides as a carbon source, which are likely more easily metabolized than the two organic acids [36,38,39,40].

#### 3.1.2. Microbial Growth

The counts (log CFU/mL) of LAB, AAB, and yeasts, as well as the production of free biomass ([X]p, in g/L) in the fermented media, can be described by the following empirical models (statistical analysis in Tables S10–S21):

LAB (24 h) = 6.63 − 0.31·A − 0.41·GW^2^ (10)

LAB (48 h) = 7.05 − 1.53·A − 0.47·GW^2^ (11)

LAB (72 h) = 6.55 − 1.36·A − 0.55·GW^2^ (12)

AAB (24 h) = 6.66 + 0.21·A − 0.48·GW^2^ (13)

AAB (48 h) = 5.57 + 0.24·A − 0.35·GW^2^ (14)

AAB (72 h) = 5.00 + 0.22·A − 0.33·GW^2^ (15)

Yeasts (24 h) = 6.80 + 0.24·A − 0.49·GW^2^ (16)

Yeasts (48 h) = 7.02 + 0.32·A − 0.42·GW^2^ (17)

Yeasts (72 h) = 7.92 + 0.37·A − 0.46·GW^2^ (18)

[X]p (24 h) = 51.23 + 3.15·A − 8.81·GW^2^ (19)

[X]p (48 h) = 51.18 + 3.20·A − 8.81·GW^2^ (20)

[X]p (72 h) = 50.52 + 2.55·A − 2.92·GW^2^ (21)

**Figure 3:**
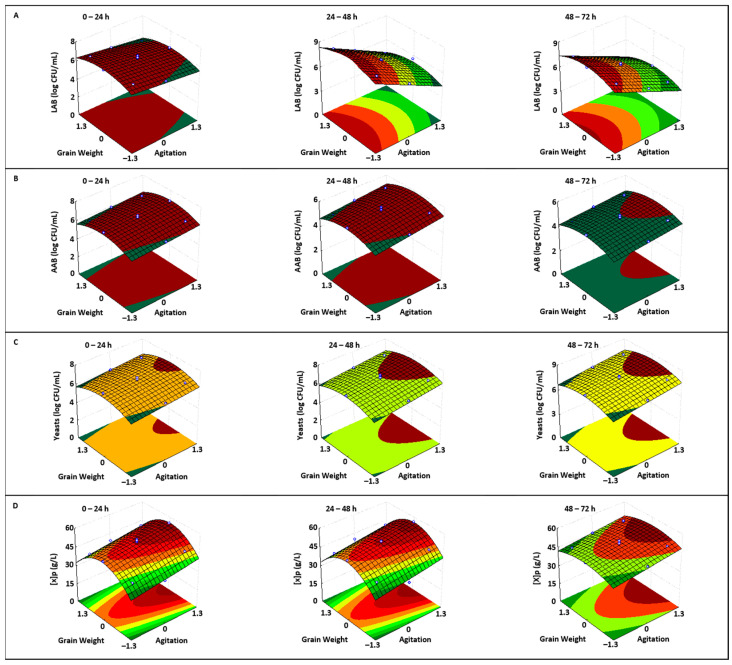
Response surfaces showing the effect of agitation and kefir grain weight on the counts (log CFU/mL) of (**A**): LAB, (**B**): AAB, (**C**): yeasts, and the production of (**D**): free biomass ([X]p) in the fermentation medium during the first (0–24 h), second (24–48 h), and third (48–72 h) subcultures. The symbols (white circles) above and below the response surfaces represent the experimental data points.

Although the optimal grain weight (1.80 g) is located at the center of the tested domain in all cases (Tables S10–S21), changes in agitation speed affected the viability of the three microbial groups and the production of free biomass in different ways. Specifically, increasing the agitation speed led to a linear decrease in the counts of LAB (Figure 3A), while the counts of AAB (Figure 3B), yeasts (Figure 3C), and free biomass production (Figure 3D) all increased.

Homolactic LAB utilize glucose and fructose as carbon sources to obtain energy for growth and maintenance, producing two moles of lactic acid and two moles of ATP per mole of glucose or fructose consumed via glycolysis (the Embden–Meyerhof–Parnas pathway) [41]. However, an increase in agitation speed can enhance oxygen dissolution in the fermentation medium, which may be detrimental to LAB growth due to their anaerobic nature [41].

The AAB population was favored by the increase in agitation speed (Figure 3B) as these bacteria are obligate aerobes [42,43]. This suggests the possible existence of competition between yeasts and AAB species for dissolved oxygen in the fermentation medium, likely favoring the yeasts, as they produced a higher concentration of biomass. However, this hypothesis requires experimental validation.

The stimulation of the yeast population by an increased agitation speed (Figure 3C) can be attributed to the enhanced dissolution of oxygen in the culture medium and the improved transfer of nutrients into cells. Increased oxygen availability and nutrient uptake promote the complete degradation of carbon sources (sugars and organic acids) through aerobic respiration, extracting a greater amount of energy stored in these nutrients. This energy can then be primarily used for biomass production and cellular maintenance [31,32,44]. Similar results were previously observed by other researchers with *Pichia stipitis* strains [44,45,46].

**Figure 4:**
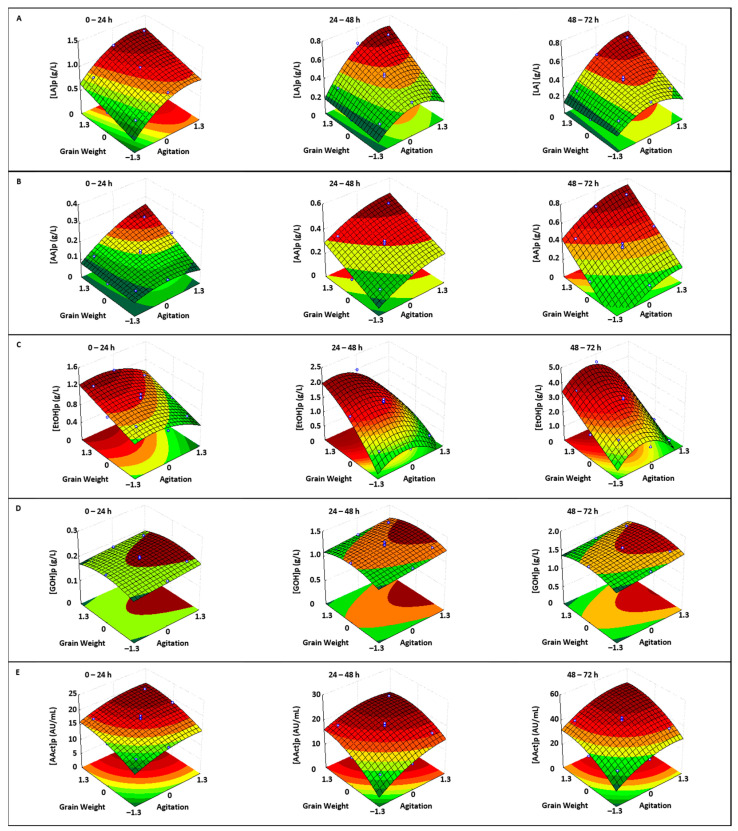
Response surfaces showing the effect of agitation and kefir grain weight on the production of (**A**): lactic acid ([LA]p), (**B**): acetic acid ([AA]c), (**C**): ethanol ([EtOH]p), (**D**): glycerol ([GOH]p) and (**E**): antibacterial activity production ([AAct]p) in the first (0–24 h), second (24–48 h), and third (48–72 h) subcultures. The symbols (white circles) above and below the response surfaces represent the experimental data points.

On the other hand, the increased viability of the three microbial populations as the GW variable rises from its minimum to optimal value appears (Figure 3A–C) to be associated with a greater transfer of microorganisms from the grains to the fermentation medium, thereby promoting their propagation. Similarly, the slight decrease in microbial counts when GW exceeds the optimal value seems to result from a dual effect. Initially, an increase in grain weight leads to a higher number of microorganisms migrating into the culture medium, which, in turn, fosters greater competition for nutrients and an increased production of inhibitory compounds such as ethanol, glycerol, lactic acid, acetic acid, and antibacterial substances (Figure 4A–E). Consequently, this results in the observed slight decrease in microbial counts (Figure 3A–C). However, the effect of independent variables (A and GW) on microbial viability was not as pronounced as their effect on nutrient (TS, CA, and CA) consumption (Figure 2A–C).

The maximum counts recorded for each microbial group in the three subcultures were as follows: LAB: 1.05 × 10^7^, 9.77 × 10^8^, and 1.91 × 10^8^ CFU/mL; AAB: 8.51 × 10^6^, 7.59 × 10^5^, and 1.91 × 10^5^ CFU/mL; and yeasts: 1.26 × 10^7^, 2.69 × 10^7^, and 2.40 × 10^8^ CFU/mL. These counts were higher (for yeasts and LAB) or very close (for AAB) to the threshold value of 10^6^ CFU/mL, which is necessary to detect stimulatory effects in the organism that consumes probiotic cells [47].

The counts of Enterobacteriaceae and *Pseudomonas* in the different kiwi fermented beverages were always below the minimum detection limit of 30 colonies, as previously observed in kefir-like beverages produced by fermenting red table grape juice with milk kefir grains [10]. This indicates that these kiwi kefir-like drinks have excellent hygienic characteristics.

Regarding free biomass production in the fermented medium (Figure 3D), it can be observed that, as expected, the independent variables (A and GW) produced the same effect on the response variable ([X]p) as they do on different microbial counts (Figure 3A–C). The maximum free biomass concentrations in the three post-incubation periods were very similar, with values of 55.21, 55.23, and 53.76 g/L, all achieved at 147 rpm and 1.80 g kefir grains (Tables S19–S21).

#### 3.1.3. Metabolite Production

The empirical equations describing the formation of lactic acid ([LA]p, in g/L), acetic ([AA]p, in g/L), ethanol ([EtOH]p, in g/L), glycerol ([GOH]p, in g/L), and antibacterial activity ([AAct]p, in AU/mL) as functions of agitation speed and kefir grain weight (Tables S22–S36) are presented below:

[LA]p (24 h) = 0.93 + 0.23·A + 0.15·GW − 0.13·A^2^ (22)

[LA]p (48 h) = 0.43 + 0.11·A + 0.10·GW + 0.06·A·GW − 0.10·A^2^ (23)

[LA]p (72 h) = 0.40 + 0.11·A + 0.06·GW + 0.07·A·GW − 0.08·A^2^ (24)

[AA]p (24 h) = 0.15 + 0.03·A + 0.03·GW + 0.03·A·GW − 0.02·A^2^ (25)

[AA]p (48 h) = 0.31 + 0.07·A + 0.09·GW − 0.02·A^2^ (26)

[AA]p (72 h) = 0.37 + 0.12·A + 0.21·GW − 0.04·A^2^ (27)

[EtOH]p (24 h) = 1.01 − 0.11·A + 0.18·GW − 0.10·A^2^ (28)

[EtOH]p (48 h) = 1.45 − 0.33·A + 0.52·GW − 0.13·A·GW − 0.32·A^2^ − 0.16·GW^2^ (29)

[EtOH]p (72 h) = 2.92 − 0.59·A + 1.07·GW − 0.99·A^2^ (30)

[GOH]p (24 h) = 0.20 + 0.01·A − 0.01·GW^2^ (31)

[GOH]p (48 h) = 1.29 + 0.06·A − 0.08·GW^2^ (32)

[GOH]p (72 h) = 1.62 + 0.08·A − 0.10·GW^2^ (33)

[AAct]p (24 h) = 18.12 + 1.54·A + 1.92·GW − 0.78·A^2^ − 0.77·GW^2^ (34)

[AAct]p (48 h) = 18.85 + 2.85·A + 3.42·GW − 0.96·A·GW – 1.39·A^2^ − 1.69·GW^2^ (35)

[AAct]p (72 h) = 41.91 + 6.38·A + 7.25·GW − 3.34·A^2^ − 3.30·GW^2^ (36)

The corresponding response surfaces generated with empirical models 22–36 are shown in Figure 4.

The maximum lactic acid production was achieved when the GW variable was consistently at its maximum value (2.81 g) and at agitation speeds (A) of 128, 134, and 143 rpm for the subcultures at 24, 48, and 72 h, respectively (Tables S22–S24; Figure 4A).

Therefore, it is reasonable to assume that by inoculating the kiwi juice with a larger amount of kefir grains, the concentration of LAB anchored to the grains increases, leading to higher lactic acid production due to the metabolic activity of these bacteria. Furthermore, lactic acid is synthesized as a mixed metabolite as its production depends on both the growth rate and biomass concentration [48]. This helps explain why agitation does not affect lactic acid production and LAB growth in the same way (Figure 3A and Figure 4A).

Acetic acid production was also maximized when the subcultures were inoculated with the maximum kefir grain weight (2.81 g) and incubated at the highest agitation speed (147 rpm) across all three subcultures (Figure 4B, Tables S25–S27). This positive effect of agitation on acetic acid production has been reported by other researchers [49,50]. In this case, the impact of agitation on acetic acid production (Figure 4B) was similar to the effect observed on AAB counts (Figure 3B).

Ethanol production was maximal in the first, second, and third kefir passages when the cultures were inoculated with the maximum grain weight and incubated at 61, 50, and 76 rpm, respectively (Tables S28–S30; Figure 4C). The decrease in ethanol production at agitation speeds higher than the optimal values is likely due to increased agitation stimulating the complete degradation of sugars by yeasts, which results in more energy being directed toward growth at the expense of ethanol production [31].

Yeasts produce glycerol to protect themselves from hyperosmotic and thermal stress. Additionally, electron-dependent glycerol formation from the central metabolic intermediate dihydroxyacetone phosphate (DHAP) can also serve as a redox sink when the regeneration of cytosolic NAD^+^ from NADH, through electron transport to oxygen in the respiratory chain, is insufficient [51,52]. It is possible that for this reason, the effect of the independent variables (GW and A) on glycerol production (Figure 4D) was similar to that observed for yeast growth (Figure 3C). In both cases, the optimal values of GW (1.80 g) and A (147 rpm) were the same (Tables S16–S18 and S31–S33).

Regarding antibacterial activity (Figure 4E), the coefficients of the quadratic terms for the independent variables in the empirical models (Tables S34–S36) indicate that the optimal values of GW and A occur at elevated levels of the independent variables: 2.79 g and 134 rpm (0–24 h); 2.44 g and 122 rpm (24–48 h); and 2.68 g and 132 rpm (48–72 h).

The antibacterial activity in the fermented medium is the sum of the contributions of the individual activities (AAct) of the different metabolites (LA, AA, EtOH, GOH, and bacteriocins) produced [53]. Therefore, the increased synthesis of lactic acid (Figure 4A), acetic acid (Figure 4B), glycerol (Figure 4D), and bacteriocins [54,55] likely counteracts the decline in ethanol production at higher agitation levels (Figure 4C).

#### 3.1.4. Comparison of Maximum Levels Achieved for Independent Variables Under Both Optimal and Cost-Effective Conditions

The growth of microbial populations in the kefir grains (LAB, AAB, yeasts, and free biomass), along with nutrient consumption and product formation, was estimated using the corresponding empirical models for the lowest agitation speed (25 rpm), which minimizes production costs, and the kefir grain supplier’s recommended GW (1.80 g/60 mL). These results were then compared to those obtained under optimal conditions for each independent variable (Table 2). This approach allows for the determination of percentage variations when cost-reducing conditions are applied.

Regarding nutrient consumption and metabolite synthesis at A = 25 rpm and GW = 1.80 g, the highest reduction percentages were observed in the consumption of total sugars (42.3–60.0%) and quinic acid (43.2–64.2%), as well as in the production of lactic acid (64.7–80.3%). In contrast, the smallest reduction percentage was observed in glycerol production (11.0–13.6%).

When comparing the microbial counts obtained at A = 25 rpm and GW = 1.8 g/60 mL to those under optimal conditions, reductions of 7.6% to 11.1% were observed in the counts of yeasts and AAB, while LAB counts remained unaffected (Table 2). This is because agitation negatively impacts LAB counts but does not significantly affect those of yeasts and AAB. Consequently, free biomass production decreased by 12.0% to 14.7%.

In summary, a culture incubated at the lowest agitation speed (A = 25 rpm) and inoculated with a GW of 1.80 g/60 mL would produce a beverage with total LAB and yeast counts greater than 10^6^ CFU/mL, high total sugar concentrations (due to the decrease in sugar consumption), and low levels of ethanol, glycerol, lactic acid, and acetic acid. Therefore, agitation appears to be necessary to reduce the total sugar content and produce a low-calorie beverage while ensuring that viable counts remain above 10^6^ CFU/mL.

#### 3.1.5. Principal Component Analysis of Kiwi Kefir-like Beverages Based on Their Microbiological and Chemical Compositions

**Figure 5:**
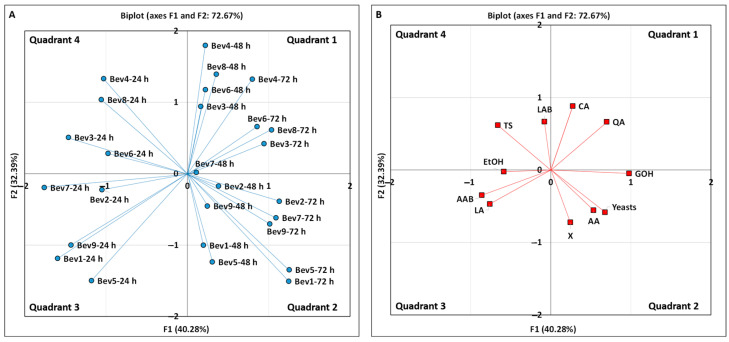
(**A**) Principal component analysis showing separation of kiwi kefir-like beverages. (**B**) Classification variables used in PCA: counts of lactic acid bacteria (LAB), acetic acid bacteria (AAB), and yeasts; concentrations of free biomass (X), total sugars (TS), citric acid (CA), quinic acid (QA), lactic acid (LA), acetic acid (AA), ethanol (EtOH), and glycerol (GOH) in fermented beverages.

A principal component analysis (PCA) was conducted to explore the relationships among 27 fermented beverages, focusing on their microbiological and chemical compositions (Table 3, Figure 5). Bartlett’s test of sphericity yielded a significance value of 0.000 (*p* < 0.050), confirming the suitability of PCA for the data.

In this analysis, the final pH and the antibacterial activity of the beverages were excluded as classification variables to prevent redundancy as the pH value is directly proportional to the concentrations of organic acids, and antibacterial activity strongly correlates with the concentrations of organic acids, alcohols, and bacteriocins produced by the kefir grains’ microbiota. This redundancy could potentially distort the interpretation of the data by overemphasizing the influence of these variables, leading to skewed or misleading results.

From the analysis, three factors (F1, F2, and F3) with eigenvalues greater than 1.0 were identified, explaining 40.28%, 32.39%, and 12.04% of the total variability, respectively (Table S37). Together, the three factors accounted for 84.72% of the total variance. The graphical representation of Factors 1 and 2 (Figure 5), which, together, explained 72.67% of the variance, shows that the beverages were grouped into four quadrants: nine in quadrant 1, nine in quadrant 2, five in quadrant 3, and four in quadrant 4 (Figure 5A). The first quadrant contained five beverages from the second passage and four from the third passage. The second quadrant included four beverages from the second passage and five from the third passage. Beverages from the first passage were primarily located in the third and fourth quadrants (Figure 5A, Table S38).

The discrimination variables were positioned in different quadrants: citric acid (CA) and quinic acid (QA) in the first quadrant; glycerol (GOH), acetic acid (AA), free biomass (X), and yeast counts in the second quadrant; ethanol (EtOH), lactic acid (LA), and AAB counts in the third quadrant; and total sugars (TS) and LAB counts in the fourth quadrant (Figure 5B, Table S39).

These results suggest that beverages from the first kefir grain passage have distinct microbiological and chemical profiles compared to drinks from the second and third passages. Specifically, the nine beverages grouped in the first quadrant (Figure 5A) exhibited the highest concentrations of citric and quinic acids (Figure 5B). Among them, Bev4-72 h and Bev8-48 h had the highest citric acid (13.91 g/L) and quinic acid (8.82 g/L) concentrations, respectively. Additionally, Bev7-48 h showed the lowest levels of citric acid (9.73 g/L) and quinic acid (7.18 g/L).

The nine beverages located in the second quadrant (Figure 5A) were grouped due to their high contents of glycerol (GOH), acetic acid (AA), free biomass (X), and viable yeasts (Figure 5B). In this group, the beverage Bev5-72 h had the highest levels of GOH (1.68 g/L) and yeast counts (8.25 log CFU/mL), while Bev5-48 h and Bev1-72 h showed the highest concentrations of free biomass (56.91 g/L) and AA (0.69 g/L), respectively (Table 3). In contrast, Bev1-48 h and Bev2-48 h exhibited the lowest concentration of GOH (1.27 g/L) among the beverages within this group. Bev1-48 h had the lowest count of yeasts (6.90 log CFU/mL), and Bev2-72 h showed the lowest AA level (0.22 g/L), while Bev7-72 h had the lowest concentration of free biomass (45.37 g/L) (Table 3).

The five beverages grouped in the third quadrant (Figure 5A) had the highest concentrations of EtOH, LA, and AAB counts (Figure 5B). In this group, Bev1-24 h, Bev5-24 h, and Bev7-24 h showed the highest levels of LA (1.31 g/L), AAB (6.93 log CFU/mL), and EtOH (4.50 g/L, corresponding to an alcohol content of 0.57%, *v*/*v*), respectively (Table 3). Bev2-24 h exhibited the lowest levels of EtOH (0.36 g/L, corresponding to an alcohol content of 0.04%, *v*/*v*) and LA (0.86 g/L), while Bev7-24 h had the lowest AAB count (5.99 log CFU/mL).

The fourth quadrant grouped four beverages with the highest levels of total sugars (TS) and LAB counts (Figure 5A,B). The highest TS concentration (107.53 g/L) and LAB count (6.86 log CFU/mL) were found in Bev4-24 h and Bev6-24 h, respectively, while Bev3-24 h and Bev8-24 h had the lowest TS concentration (90.70 g/L) and LAB count (6.01 log CFU/mL) within this group.

In general, all the beverages have a low ethanol content, ranging from 0.04 to 0.57% (*v*/*v*), and a low glycerol content, ranging from 0.01 to 0.13%, (*v*/*v*). These levels are significantly lower than the alcohol content in wines commonly sold in Spain, which typically ranges from 11.00 to 14.30% (*v*/*v*) [10]. Therefore, the 27 kiwi kefir-like beverages can be classified as low-alcohol drinks. Additionally, these beverages are acidic, with pH values ranging from 2.98 to 3.21 (Table 3). The kiwi juice used as the substrate has a pH value of 3.27 ± 0.17, which slightly changes due to the production and consumption of organic acids by the microbiota present in the kefir grains [11]. Consequently, analyzing the concentrations of sugars, as well as the counts of bacteria and yeasts, is crucial for selecting a beverage suitable for human consumption.

A detailed analysis of the experimental data (Table 3 and Figure 5) revealed that the beverages Bev4-24 h and Bev6-24 h contained the highest concentrations of total sugars at 107.53 and 102.66 g/L, respectively. These values represent 87.02% and 83.09% of the initial total sugar concentration in the unfermented kiwi juice. This suggests that both Bev4-24 h and Bev6-24 h are high-calorie drinks, and their consumption could lead to excessive calorie intake. Notably, both beverages also contained high counts of LAB, AAB, and yeasts (greater than 10^6^ CFU/mL), except for the LAB count in Bev2-24 h, which was slightly below 10^6^ CFU/mL.

In contrast, Bev7-72 h and Bev1-72 h can be considered the lowest-calorie drinks as they contain the lowest total sugar content (31.13 and 34.13 g/L, respectively), representing 25.19% and 27.62% of the initial TS concentration in the unfermented kiwi juice. However, regarding the microbial counts, only the yeast count in both beverages exceeded 10^6^ CFU/mL (Table 3).

The counts of LAB, AAB, and yeasts in beverages Bev5-24 h and Bev9-24 h were higher than 10^6^ CFU/mL, and their total sugars levels were 72.37 and 71.40 g/L, respectively, representing 58.57% and 57.79% of the initial TS concentration in the unfermented kiwi juice.

Additionally, ten kefir-like drinks (Bev8-24 h, Bev3-48 h, Bev4-48 h, Bev6-48 h, Bev7-48 h, Bev8-48 h, Bev9-48 h, Bev3-72 h, Bev6-72 h, and Bev9-72 h) had counts of LAB and yeasts, the microbial groups with a recognized probiotic effect [8,16], exceeding 10^6^ CFU/mL. Among these ten beverages, Bev9-72 h, Bev7-48 h, and Bev3-72 h were the lowest in calories, containing 45.86, 53.89, and 56.56 g TS/L, respectively, which represent 37.12%, 43.61%, and 45.77% of the initial TS concentration present in the substrate.

In summary, Bev7-48 h (A = 86 rpm, GW = 2.81 g), Bev3-72 h (A = 38 rpm, GW = 2.60 g), and Bev9-72 h (A = 86 rpm, GW = 1.80 g) could be selected as suitable, potentially probiotic, low-alcohol, and low-calorie drinks.

Since these three beverages were produced using agitation speeds of 38 rpm (Bev3-72 h) and 86 rpm (Bev7-48 h and Bev9-72 h), it can be suggested that these low speeds are suitable for producing beverages with potentially probiotic properties and a low total sugar content. Regarding the weight of the kefir grains, it is important to note that the effect of this variable depends on the number of kefir grain passages in the kiwi juice. For example, Bev7-48 h was produced during the second kefir grain passage with a grain weight of 2.81 g. In contrast, Bev3-72 h and Bev9-72 h were produced during the third kefir grain passage, with lower grain weights of 2.60 g and 1.80 g, respectively.

However, in addition to these characteristics (a low alcohol content, low calories, and probiotic cell counts higher than 10^6^ CFU/mL), a probiotic beverage should also possess an aromatic profile that ensures consumer acceptance, as well as good stability during refrigerated storage. Our research team is currently conducting studies on these aspects.

## 4. Conclusions

The results obtained in this study enabled the selection of low-alcohol and low-calorie kiwi kefir-like beverages with probiotic cell counts exceeding 10^6^ CFU/mL and good hygienic properties. These beverages were produced through 24 h batch fermentations across three kefir grain passages, following a second-order orthogonal factorial design using the kefir grain weight (GW) and agitation speed (A) as independent variables. This approach allowed for the identification of optimal GW and A values to maximize sugar consumption and microbial growth (mainly LAB and yeasts) while minimizing the production of alcohols and organic acids.

From a practical standpoint, this study may support the valorization of small-caliber kiwifruits, which typically have low commercial value, by contributing to the cost-effective production of functional kiwi kefir-like beverages. This could also help increase the income of kiwifruit producers.

Furthermore, ongoing research in our laboratory, as a continuation of this work, focuses on the characterization of volatile compounds in the various kiwifruit kefir-like beverages produced in this study. This will help identify beverages with optimal chemical and microbiological characteristics and a broader aromatic profile. Additionally, the microbial and chemical stability of the most suitable beverages during refrigerated storage will be further investigated.

## Figures and Tables

**Table 1:** Experimental domain and natural and coded values of agitation speeds (A) and kefir grain weight (GW) in second-order orthogonal factorial design. Bev: beverage.

Points	Codified Values		Natural Values		
	A	GW	A (rpm)	GW (g)	Designation
Factorial	1	1	134	2.60	Bev1
	1	−1	134	1.00	Bev2
	−1	1	38	2.60	Bev3
	−1	−1	38	1.00	Bev4
Axial	1.267	0	147	1.80	Bev5
	−1.267	0	25	1.80	Bev6
	0	1.267	86	2.81	Bev7
	0	−1.267	86	0.79	Bev8
* Center	0	0	86	1.80	Bev9

* Five replicates were performed at the center of the domain.

**Table 2:** Comparison of maximum levels (*L_max_*) calculated for different culture variables under optimal agitation and grain weight conditions and estimated levels (*L_est_*) for minimum agitation (A = 25 rpm) and GW recommended by supplier of kefir grains (GW = 1.80 g/60 mL), both obtained with corresponding empirical models for each variable.

Variable *	*L_max_*			*L_est_*			$\%Red=\frac{L_{max}-L_{est}}{L_{max}}\cdot 100$		
	24 h	48 h	72 h	24 h	48 h	72 h	24 h	48 h	72 h
[TS]c (g/L)	57.23	73.43	93.48	23.32	29.34	53.97	59.2	60.0	42.3
[CA]c (g/L)	13.83	12.40	11.62	9.16	8.15	7.84	33.8	34.3	32.5
[QA]c (g/L)	4.37	1.70	3.02	2.48	0.79	1.08	43.2	53.5	64.2
[LA]p (g/L)	1.22	0.66	0.60	0.43	0.13	0.14	64.7	80.3	76.7
[AA]p (g/L)	0.26	0.47	0.72	0.08	0.18	0.15	69.2	61.7	79.2
[EtOH]p (g/L)	2.04	4.36	1.27	1.36	2.06	1.00	33.3	52.7	21.3
[GOH]p (g/L)	0.22	1.36	1.71	0.19	1.21	1.52	13.6	11.0	11.1
[AAct]p (AU/mL)	20.02	20.92	48.60	14.92	13.02	28.47	25.5	37.8	41.4
LAB (log(CFU/mL))	7.02	8.99	8.28	7.02	8.99	8.28	0	0	0
AAB (log(CFU/mL))	6.93	5.88	5.28	6.40	5.26	4.73	7.6	10.5	10.4
Yeasts (log(CFU/mL))	7.10	7.43	8.38	6.50	6.61	7.42	8.4	11.0	11.1
[X]p (g/L)	55.21	55.23	53.76	47.24	47.13	47.29	14.4	14.7	12.0

* Consumptions of total sugar ([TS]c), citric acid ([CA]c), and quinic acid ([QA]c) and productions of lactic acid ([LA]p), acetic acid ([AA]p), ethanol ([EtOH]p), glycerol ([GOH]p), free biomass ([X]p), and antibacterial activity ([AAct]p). Counts of LAB, AAB, and yeasts in log(CFU/mL). %Red: reduction (%).

**Table 3:** Microbiological and chemical compositions of the 27 beverages obtained after the first, second, and third kefir grain passages.

Beverage	Passage	LAB *	AAB *	Yeasts *	[X] *	[TS] *	[CA] *	[QA] *	[LA] *	[AA] *	[EtOH] *	[GOH] *	pH
Be1-24 h	1st	5.85	6.42	6.64	45.71	71.10	5.60	5.51	1.31	0.22	2.32	0.20	3.19
Be2-24 h	1st	5.80	6.43	6.66	45.69	87.14	8.88	6.58	0.86	0.09	0.36	0.20	3.11
Be3-24 h	1st	6.51	5.92	5.96	39.81	90.71	9.87	6.90	0.78	0.12	3.50	0.18	3.08
Be4-24 h	1st	6.53	5.88	5.92	39.64	107.53	13.02	7.92	0.49	0.09	2.11	0.18	3.01
Be5-24 h	1st	6.22	6.93	7.09	55.79	72.37	5.55	5.49	1.02	0.19	0.65	0.21	3.21
Be6-24 h	1st	6.86	6.57	6.88	47.32	102.66	8.86	7.32	0.41	0.07	1.66	0.20	3.12
Be7-24 h	1st	5.94	5.99	6.06	39.28	75.78	8.25	6.37	1.15	0.18	4.50	0.18	3.11
Be8-24 h	1st	6.01	5.92	6.13	34.76	98.73	12.54	7.77	0.89	0.11	1.04	0.18	3.00
Be9-24 h	1st	6.63	6.66	6.79	51.23	71.40	5.88	5.92	0.93	0.16	2.94	0.20	3.20
Be1-48 h	2nd	5.12	5.46	6.90	46.15	49.66	7.52	6.56	0.65	0.44	0.99	1.27	3.13
Be2-48 h	2nd	5.06	5.48	6.92	46.97	65.63	10.26	7.43	0.33	0.25	0.66	1.27	3.07
Be3-48 h	2nd	8.27	4.92	6.20	40.56	76.37	11.08	7.56	0.30	0.35	1.22	1.14	3.04
Be4-48 h	2nd	8.31	4.87	6.15	40.18	92.32	13.72	8.70	0.22	0.15	0.96	1.13	2.99
Be5-48 h	2nd	5.66	5.75	7.27	56.91	65.26	6.48	6.55	0.42	0.38	0.70	1.34	3.17
Be6-48 h	2nd	9.32	5.28	6.60	48.47	94.38	10.09	8.49	0.14	0.13	0.92	1.21	3.07
Be7-48 h	2nd	6.36	5.04	6.30	40.37	53.89	9.73	7.18	0.64	0.40	1.26	1.16	3.06
Be8-48 h	2nd	6.46	4.99	6.37	33.46	76.22	13.32	8.82	0.37	0.21	0.69	1.17	2.98
Be9-48 h	2nd	7.03	5.57	7.02	51.20	65.82	7.08	7.37	0.43	0.31	1.02	1.29	3.14
Be1-72 h	3rd	4.28	4.88	7.84	49.73	34.13	8.04	7.71	0.62	0.69	0.99	1.60	3.07
Be2-72 h	3rd	4.77	4.90	7.86	49.89	54.93	10.64	8.15	0.34	0.22	0.35	1.60	3.05
Be3-72 h	3rd	7.31	4.39	7.03	44.64	56.56	11.42	8.29	0.26	0.44	1.82	1.43	**3.02**
Be4-72 h	3rd	7.40	4.35	6.98	44.29	71.63	13.91	8.72	0.25	0.00	0.65	1.42	2.99
Be5-72 h	3rd	4.75	5.14	8.25	52.34	44.67	7.06	7.70	0.38	0.45	0.46	1.68	3.12
Be6-72 h	3rd	8.04	4.72	7.49	46.26	68.00	9.96	8.64	0.14	0.15	1.46	1.52	3.07
Be7-72 h	3rd	5.40	4.51	7.15	45.37	31.13	10.14	8.07	0.51	0.64	1.98	1.46	3.02
Be8-72 h	3rd	5.89	4.45	7.23	45.89	58.88	13.53	8.65	0.36	0.15	0.44	1.47	2.98
Be9-72 h	3rd	6.55	5.00	7.92	50.56	45.86	7.63	8.09	0.41	0.37	1.45	1.62	3.10

* Counts of LAB, AAB, and yeasts in log (CFU/mL). Concentrations of free biomass [X], total sugars [TS], citric acid [CA], quinic acid [QA], lactic acid [LA], acetic acid [AA], ethanol [EtOH], and glycerol [GOH] in g/L.

## Data Availability

The data supporting the findings of this study are available within the article and its Supplementary Materials. Further inquiries can be directed to the corresponding author.

## Supplementary Materials

The following supporting information can be downloaded at https://www.mdpi.com/article/10.3390/foods14101681/s1, Table S1: The results of the experimental design and analysis of the significance of the proposed model for total sugar ([TS]c, as the sum of glucose and fructose) consumption at 24 h of fermentation. Table S2: The results of the experimental design and analysis of the significance of the proposed model for total sugar ([TS]c, as the sum of glucose and fructose) consumption at 48 h of fermentation. Table S3: The results of the experimental design and analysis of the significance of the proposed model for total sugar ([TS]c, as the sum of glucose and fructose) consumption at 72 h of fermentation. Table S4: The results of the experimental design and analysis of the significance of the proposed model for citric acid consumption ([CA]c) at 24 h of fermentation. Table S5: The results of the experimental design and analysis of the significance of the proposed model for citric acid consumption ([CA]c) at 48 h of fermentation. Table S6: The results of the experimental design and analysis of the significance of the proposed model for citric acid consumption ([CA]c) at 72 h of fermentation. Table S7: The results of the experimental design and analysis of the significance of the proposed model for quinic acid consumption ([QA]c) at 24 h of fermentation. Table S8: The results of the experimental design and analysis of the significance of the proposed model for quinic acid consumption ([QA]c) at 48 h of fermentation. Table S9: The results of the experimental design and analysis of the significance of the proposed model for quinic acid consumption ([QA]c) at 72 h of fermentation. Table S10: The results of the experimental design and analysis of the significance of the proposed model for lactic acid bacteria (LAB) counts at 24 h of fermentation. Table S11: The results of the experimental design and analysis of the significance of the proposed model for lactic acid bacteria (LAB) counts at 48 h of fermentation. Table S12: The results of the experimental design and analysis of the significance of the proposed model for lactic acid bacteria (LAB) counts at 72 h of fermentation. Table S13: The results of the experimental design and analysis of the significance of the proposed model for acetic acid bacteria (AAB) counts at 24 h of fermentation. Table S14: The results of the experimental design and analysis of the significance of the proposed model for acetic acid bacteria (AAB) counts at 48 h of fermentation. Table S15: The results of the experimental design and analysis of the significance of the proposed model for acetic acid bacteria (AAB) counts at 72 h of fermentation. Table S16: The results of the experimental design and analysis of the significance of the proposed model for yeast counts at 24 h of fermentation. Table S17: The results of the experimental design and analysis of the significance of the proposed model for yeast counts at 48 h of fermentation. Table S18: The results of the experimental design and analysis of the significance of the proposed model for yeast counts at 72 h of fermentation. Table S19: The results of the experimental design and analysis of the significance of the proposed model for free biomass production ([X]p) at 24 h of fermentation. Table S20: The results of the experimental design and analysis of the significance of the proposed model for free biomass production ([X]p) at 48 h of fermentation. Table S21: The results of the experimental design and analysis of the significance of the proposed model for free biomass production ([X]p) at 72 h of fermentation. Table S22: The results of the experimental design and analysis of the significance of the proposed model for lactic acid production ([LA]p) at 24 h of fermentation. Table S23: The results of the experimental design and analysis of the significance of the proposed model for lactic acid production ([LA]p) at 48 h of fermentation. Table S24: The results of the experimental design and analysis of the significance of the proposed model for lactic acid production ([LA]p) at 72 h of fermentation. Table S25: The results of the experimental design and analysis of the significance of the proposed model for acetic acid production ([AA]p) at 24 h of fermentation. Table S26: The results of the experimental design and analysis of the significance of the proposed model for acetic acid production ([AA]p) at 48 h of fermentation. Table S27: The results of the experimental design and analysis of the significance of the proposed model for acetic acid production ([AA]p) at 72 h of fermentation. Table S28: The results of the experimental design and analysis of the significance of the proposed model for ethanol production ([EtOH]p) at 24 h of fermentation. Table S29: The results of the experimental design and analysis of the significance of the proposed model for ethanol production ([EtOH]p) at 48 h of fermentation. Table S30: The results of the experimental design and analysis of the significance of the proposed model for ethanol production ([EtOH]p) at 72 h of fermentation. Table S31: The results of the experimental design and analysis of the significance of the proposed model for glycerol production ([GOH]p) at 24 h of fermentation. Table S32: The results of the experimental design and analysis of the significance of the proposed model for glycerol production ([GOH]p) at 48 h of fermentation. Table S33: The results of the experimental design and analysis of the significance of the proposed model for glycerol production ([GOH]p) at 72 h of fermentation. Table S34: The results of the experimental design and analysis of the significance of the proposed model for antibacterial activity production ([AAct]p) at 24 h of fermentation. Table S35: The results of the experimental design and analysis of the significance of the proposed model for antibacterial activity production ([AAct]p) at 48 h of fermentation. Table S36: The results of the experimental design and analysis of the significance of the proposed model for antibacterial activity production ([AAct]p) at 72 h of fermentation. Table S37: Eigenvalues and percentage of explained variance by each component. Table S38: The correlation of beverages with the factors of the PCA based on factor loadings. Table S39: Correlation of microbiological and physico-chemical variables with factors of PCA based on factor loadings.
