# Supplementary material for: Microbiological and Chemical Profiles of Kiwi Kefir-like Beverages Produced Using Different Agitation Speeds and Kefir Grain Weights
Source: Foods. 2025 May 9;14(10):1681. doi: 10.3390/foods14101681 (PMC12111302; doi:10.3390/foods14101681)
Supplement: Supplementary file 1 [file foods-14-01681-s001.zip › foods-3613453-supplementary.pdf]

Table S1. Results of the experimental design and analysis of the significance of the proposed model for total sugars ([TS]c, as the sum of glucose and fructose) consumption at 24 h of fermentation. Y: response (g/L); $\hat{Y}$: expected response (g/L); NS: non-significant coefficient; SS: sum of squares; df: degrees of freedom; QM: quadratic means; M: model; E: total error; Ee: experimental error; LF: lack of fit; Var(Ee): variance of the error experimental. A: agitation speed; GW: kefir grains weight.

A	GW	Y	$\hat{Y}$	Coefficients	*t*	Model	
1	1	52.46	53.06	51.99	17.48	51.99	
1	-1	36.42	35.88	10.87	4.35	10.87	A
-1	1	32.85	31.32	8.59	3.44	8.59	GW
-1	-1	16.03	14.14	-0.24	0.06	NS	A·GW
1.267	0	51.19	50.87	-9.28	3.14	-9.28	A^2
-1.267	0	20.90	23.32	-9.11	3.08	-9.11	GW^2
0	1.267	47.78	48.24	Mean response = 41.79			
0	-1.267	24.83	26.47	Central mean response = 52.16			
0	0	48.40	51.99	Var(Ee) = 45.09			
0	0	62.98	51.99	t ($\alpha < 0.05$; $df = 4$) = 2.78			
0	0	45.15	51.99	QMM/QME = 23.02	$F_8^4(\alpha = 0.05) = 3.84$		
0	0	52.18	51.99	QMLF/QMM = 0.50	$F_4^8(\alpha = 0.05) = 6.04$		
0	0	52.07	51.99	QME/QMEe = 0.54	$F_4^8(\alpha = 0.05) = 6.04$		
	SS	df	QM	QMLF/QMEe = 0.09	$F_4^4(\alpha = 0.05) = 6.39$		
Model	2256.04	4	564.01	$r^2 = 0.920$	adjusted $r^2 = 0.880$		
Error	195.97	8	24.50				
Ee	180.35	4	45.09	Optimum A value = 124 rpm			
LF	15.62	4	3.90	Optimum GW = 2.18 g			
Total	2452.01	12	204.33	Maximum [TS]c = 57.20 g/L			

Table S2. Results of the experimental design and analysis of the significance of the proposed model for total sugars ([TS]c, as the sum of glucose and fructose) consumption at 48 h of fermentation. Y: response (g/L); Ŷ: expected response (g/L); NS: non-significant coefficient; SS: sum of squares; df: degrees of freedom; QM: quadratic means; M: model; E: total error; Ee: experimental error; LF: lack of fit; Var(Ee): variance of the error experimental. A: agitation speed; GW: kefir grains weight.

A	GW	Y	Ŷ	Coefficients	*t*	Model	
1	1	73.90	71.57	57.51	47.82	57.51	
1	-1	57.93	54.87	12.52	12.39	12.52	A
-1	1	47.19	46.53	8.35	8.26	8.35	GW
-1	-1	31.24	29.83	0.00	0.00	NS	A·GW
1.267	0	58.30	61.93	-7.66	6.41	-7.66	A^2
-1.267	0	29.18	30.20	1.54	1.29	NS	GW^2
0	1.267	69.67	68.94	Mean response = 54.11			
0	-1.267	47.34	47.78	Central mean response = 57.74			
0	0	55.78	58.36	Var(Ee) = 7.37			
0	0	61.88	58.36	t (α < 0.05; df = 4) = 2.78			
0	0	57.67	58.36	QMM/QME = 91.38	$F_9^3(\alpha = 0.05) = 3.86$		
0	0	58.44	58.36	QMLF/QMM = 0.38	$F_3^8(\alpha = 0.05) = 8.85$		
0	0	54.91	58.36	QME/QMEe = 0.96	$F_4^9(\alpha = 0.05) = 6.00$		
	SS	df	QM	QMLF/QMEe = 0.93	$F_4^5(\alpha = 0.05) = 6.26$		
Model	1936.02	3	645.34	r^2 = 0.968	adjusted r^2 = 0.958		
Error	63.56	9	7.06				
Ee	29.46	4	7.37	Optimum A value = 130 rpm			
LF	34.10	5	6.82	Optimum GW = 2.81 g			
Total	949.58	12	166.63	Maximum [TS]c = 61.08 g/L			

Table S3. Results of the experimental design and analysis of the significance of the proposed model for total sugars ([TS]c, as the sum of glucose and fructose) consumption at 72 h of fermentation. Y: response (g/L); Ŷ: expected response (g/L); NS: non-significant coefficient; SS: sum of squares; df: degrees of freedom; QM: quadratic means; M: model; E: total error; Ee: experimental error; LF: lack of fit; Var(Ee): variance of the error experimental. A: agitation speed; GW: kefir grains weight.

A	GW	Y	Ŷ	Coefficients	*t*	Model	
1	1	89.43	89.71	77.88	95.15	77.88	
1	-1	68.63	70.00	9.52	13.84	9.52	A
-1	1	67.00	70.66	9.85	14.32	9.85	GW
-1	-1	51.93	50.95	1.43	1.55	NS	A·GW
1.267	0	78.89	77.94	-7.37	9.06	-7.37	A^2
-1.267	0	55.56	53.80	-0.31	0.39	NS	GW^2
0	1.267	92.43	90.19	Mean response = 73.62			
0	-1.267	64.68	65.22	Central mean response = 77.70			
0	0	75.29	77.71	Var(Ee) = 3.41			
0	0	76.83	77.71	t (α < 0.05; df = 4) = 2.78			
0	0	80.04	77.71	QMM/QME = 124.83		$F_9^3(\alpha = 0.05) = 3.86$	
0	0	77.39	77.71	QMLF/QMM = 0.38		$F_3^8(\alpha = 0.05) = 8.85$	
0	0	78.93	77.71	QME/QMEe = 1.28		$F_4^9(\alpha = 0.05) = 6.00$	
	SS	df	QM	QMLF/QMEe = 1.50		$F_4^5(\alpha = 0.05) = 6.26$	
Model	1634.52	3	544.84	r^2 = 0.977		adjusted r^2 = 0.969	
Error	39.28	9	4.36				
Ee	13.65	4	3.41	Optimum A value = 125 rpm			
LF	25.63	5	5.13	Optimum GW = 2.81 g			
Total	1673.81	12	139.48	Maximum [TS]c = 93.44 g/L			

Table S4. Results of the experimental design and analysis of the significance of the proposed model for citric acid consumption ([CA]c) at 24 h of fermentation. Y: response (g/L); Ŷ: expected response (g/L); NS: non-significant coefficient; SS: sum of squares; df: degrees of freedom; QM: quadratic means; M: model; E: total error; Ee: experimental error; LF: lack of fit; Var(Ee): variance of the error experimental. A: agitation speed; GW: kefir grains weight.

A	GW	Y	Ŷ	Coefficients	*t*	Model	
1	1	11.67	11.60	11.37	24.28	11.37	
1	-1	8.39	8.30	1.75	4.44	1.75	A
-1	1	7.40	8.10	1.65	4.18	1.65	GW
-1	-1	4.25	4.81	0.03	0.06	NS	A·GW
1.267	0	11.72	13.16	-0.76	1.64	NS	A^2
-1.267	0	8.41	8.74	-2.75	5.90	-2.75	GW^2
0	1.267	9.02	8.62	Mean response = 9.43			
0	-1.267	4.73	4.45	Central mean response = 11.39			
0	0	10.80	10.95	Var(Ee) = 1.12			
0	0	12.91	10.95	t ($\alpha < 0.05$; $df = 4$) = 2.78			
0	0	10.07	10.95	QMM/QME = 27.92		$F_9^3(\alpha = 0.05) = 3.86$	
0	0	11.59	10.95	QMLF/QMM = 0.39		$F_3^8(\alpha = 0.05) = 8.85$	
0	0	11.56	10.95	QME/QMEe = 0.86		$F_4^9(\alpha = 0.05) = 6.00$	
	SS	df	QM	QMLF/QMEe = 0.75		$F_4^5(\alpha = 0.05) = 6.26$	
Model	80.53	3	26.84	$r^2 = 0.903$		adjusted $r^2 = 0.871$	
Error	8.65	9	0.96				
Ee	4.47	4	1.12	Optimum A value = 147 rpm			
LF	4.18	5	0.84	Optimum GW = 2.04 g			
Total	89.19	12	7.43	Maximum [CA]c = 13.83 g/L			

Table S5. Results of the experimental design and analysis of the significance of the proposed model for citric acid consumption ([CA]c) at 48 h of fermentation. Y: response (g/L); $\hat{Y}$: expected response (g/L); NS: non-significant coefficient; SS: sum of squares; df: degrees of freedom; QM: quadratic means; M: model; E: total error; Ee: experimental error; LF: lack of fit; Var(Ee): variance of the error experimental. A: agitation speed; GW: kefir grains weight.

A	GW	Y	$\hat{Y}$	Coefficients	*t*	Model	
1	1	9.75	9.97	10.19	35.70	10.19	
1	-1	7.01	7.22	1.61	6.70	1.61	A
-1	1	6.19	6.75	1.38	5.74	1.38	GW
-1	-1	3.55	4.00	0.03	0.08	NS	A·GW
1.267	0	10.79	11.80	-0.76	2.69	NS	A^2
-1.267	0	7.18	7.73	-2.78	9.81	-2.78	GW^2
0	1.267	7.54	7.04	Mean response = 8.22			
0	-1.267	3.95	3.56	Central mean response = 10.19			
0	0	9.59	9.77	Var(Ee) = 0.41			
0	0	9.56	9.77	t ($\alpha < 0.05$; $df = 4$) = 2.78			
0	0	10.80	9.77	QMM/QME = 44.52	$F_9^3(\alpha = 0.05) = 3.86$		
0	0	10.91	9.77	QMLF/QMM = 0.39	$F_3^8(\alpha = 0.05) = 8.85$		
0	0	10.07	9.77	QME/QMEe = 1.30	$F_4^9(\alpha = 0.05) = 6.00$		
	SS	df	QM	QMLF/QMEe = 1.54	$F_4^5(\alpha = 0.05) = 6.26$		
Model	72.23	3	24.08	$r^2 = 0.937$	adjusted $r^2 = 0.916$		
Error	4.87	9	0.54				
Ee	1.66	4	0.42	Optimum A value = 147 rpm			
LF	3.21	5	0.64	Optimum GW = 2.00 g			
Total	77.10	12	6.43	Maximum [CA]c = 12.40 g/L			

Table S6. Results of the experimental design and analysis of the significance of the proposed model for citric acid consumption ([CA]c) at 72 h of fermentation. Y: response (g/L); $\hat{Y}$: expected response (g/L); NS: non-significant coefficient; SS: sum of squares; df: degrees of freedom; QM: quadratic means; M: model; E: total error; Ee: experimental error; LF: lack of fit; Var(Ee): variance of the error experimental. A: agitation speed; GW: kefir grains weight.

A	GW	Y	$\hat{Y}$
1	1	9.23	9.36
1	-1	6.63	6.75
-1	1	5.85	6.49
-1	-1	3.36	3.89
1.267	0	10.21	11.12
-1.267	0	7.31	7.50
0	1.267	7.13	6.65
0	-1.267	3.74	3.35
0	0	10.32	9.31
0	0	9.52	9.31
0	0	9.08	9.31
0	0	9.04	9.31
0	0	10.22	9.31

Coefficients	*t*	Model	
9.65	35.74	9.65	
1.43	6.31	1.43	A
1.30	5.74	1.30	GW
0.02	0.08	NS	A·GW
-0.62	2.30	NS	A^2
-2.69	10.01	-2.69	GW^2

Mean response = 7.82

Central mean response = 9.64

Var(Ee) = 0.37

t ($\alpha < 0.05$; $df = 4$) = 2.78

QMM/QME = 48.21	$F_9^3(\alpha = 0.05) = 3.86$
QMLF/QMM = 0.39	$F_3^8(\alpha = 0.05) = 8.85$
QME/QMEe = 1.20	$F_4^9(\alpha = 0.05) = 6.00$
QMLF/QMEe = 1.35	$F_4^5(\alpha = 0.05) = 6.26$
$r^2 = 0.941$	adjusted $r^2 = 0.922$

	SS	df	QM
Model	64.25	3	21.42
Error	4.00	9	0.44
Ee	1.49	4	0.37
LF	2.51	5	0.50
Total	68.25	12	5.69

Optimum A value = 147 rpm

Optimum GW = 1.99 g

Maximum [CA]c = 11.62 g/L

Table S7. Results of the experimental design and analysis of the significance of the proposed model for quinic acid consumption ([QA]c) at 24 h of fermentation. Y: response (g/L); Ŷ: expected response (g/L); NS: non-significant coefficient; SS: sum of squares; df: degrees of freedom; QM: quadratic means; M: model; E: total error; Ee: experimental error; LF: lack of fit; Var(Ee): variance of the error experimental. A: agitation speed; GW: kefir grains weight.

A	GW	Y	Ŷ	Coefficients	*t*	Model	
1	1	3.79	3.83	3.37	29.34	3.37	
1	-1	2.72	2.76	0.70	7.27	0.70	A
-1	1	2.40	2.43	0.54	5.57	0.54	GW
-1	-1	1.38	1.36	0.01	0.10	NS	A·GW
1.267	0	3.81	4.13	-0.23	2.01	NS	A^2
-1.267	0	1.98	2.35	-0.64	5.64	-0.64	GW^2
0	1.267	2.93	2.89	Mean response = 2.88			
0	-1.267	1.53	1.53	Central mean response = 3.38			
0	0	3.28	3.24	Var(Ee) = 0.07			
0	0	3.51	3.24	t ($\alpha < 0.05$; $df = 4$) = 2.78			
0	0	3.76	3.24	QMM/QME = 37.92		$F_9^3(\alpha = 0.05) = 3.86$	
0	0	3.09	3.24	QMLF/QMM = 0.39		$F_3^8(\alpha = 0.05) = 8.85$	
0	0	3.27	3.24	QME/QMEe = 1.02		$F_4^9(\alpha = 0.05) = 6.00$	
	SS	df	QM	QMLF/QMEe = 1.03		$F_4^5(\alpha = 0.05) = 6.26$	
Model	7.71	3	2.57	$r^2 = 0.927$		adjusted $r^2 = 0.902$	
Error	0.62	9	0.07				
Ee	0.27	4	0.07	Optimum A value = 147 rpm			
LF	0.35	5	0.07	Optimum GW = 2.13 g			
Total	8.33	12	0.69	Maximum [QA]c = 4.37 g/L			

Table S8. Results of the experimental design and analysis of the significance of the proposed model for quinic acid consumption ([QA]c) at 48 h of fermentation. Y: response (g/L); Ŷ: expected response (g/L); NS: non-significant coefficient; SS: sum of squares; df: degrees of freedom; QM: quadratic means; M: model; E: total error; Ee: experimental error; LF: lack of fit; Var(Ee): variance of the error experimental. A: agitation speed; GW: kefir grains weight.

A	GW	Y	Ŷ	Coefficients	*t*	Model	
1	1	2.74	2.85	1.91	38.06	1.91	
1	-1	1.87	1.71	0.66	15.55	0.66	A
-1	1	1.74	1.53	0.57	13.43	0.57	GW
-1	-1	0.60	0.40	-0.07	1.19	NS	A·GW
1.267	0	2.75	2.75	0.01	0.13	NS	A^2
-1.267	0	0.81	1.08	-0.29	5.88	-0.29	GW^2
0	1.267	2.12	2.16	Mean response = 1.75			
0	-1.267	0.48	0.73	Central mean response = 1.93			
0	0	1.97	1.92	Var(Ee) = 0.01			
0	0	2.09	1.92	t ($\alpha < 0.05$; $df = 4$) = 2.78			
0	0	1.80	1.92	QMM/QME = 56.91		$F_9^3(\alpha = 0.05) = 3.86$	
0	0	1.85	1.92	QMLF/QMM = 0.39		$F_3^8(\alpha = 0.05) = 8.85$	
0	0	1.96	1.92	QME/QMEe = 2.68		$F_4^9(\alpha = 0.05) = 6.00$	
	SS	df	QM	QMLF/QMEe = 4.02		$F_4^5(\alpha = 0.05) = 6.26$	
Model	5.87	3	1.96	$r^2 = 0.950$		adjusted $r^2 = 0.933$	
Error	0.31	9	0.03				
Ee	0.05	4	0.01	Optimum A value = 147 rpm			
LF	0.26	5	0.05	Optimum GW = 2.57 g			
Total	6.18	12	0.52	Maximum [QA]c = 3.02 g/L			

Table S9. Results of the experimental design and analysis of the significance of the proposed model for quinic acid consumption ([QA]c) at 72 h of fermentation. Y: response (g/L); Ŷ: expected response (g/L); NS: non-significant coefficient; SS: sum of squares; df: degrees of freedom; QM: quadratic means; M: model; E: total error; Ee: experimental error; LF: lack of fit; Var(Ee): variance of the error experimental. A: agitation speed; GW: kefir grains weight.

A	GW	Y	Ŷ	Coefficients	*t*	Model	
1	1	1.59	1.60	1.20	47.55	1.20	
1	-1	1.15	1.15	0.32	15.31	0.32	A
-1	1	1.01	0.96	0.22	10.61	0.22	GW
-1	-1	0.58	0.51	0.00	0.15	NS	A·GW
1.267	0	1.60	1.60	-0.02	0.63	NS	A^2
-1.267	0	0.66	0.78	-0.14	5.39	-0.14	GW^2
0	1.267	1.23	1.26	Mean response = 1.12			
0	-1.267	0.65	0.69	Central mean response = 1.21			
0	0	1.19	1.19	Var(Ee) = 0.003			
0	0	1.19	1.19	t (α < 0.05; df = 4) = 2.78			
0	0	1.26	1.19	QMM/QME = 91.81	$F_9^3(\alpha = 0.05) = 3.86$		
0	0	1.26	1.19	QMLF/QMM = 0.38	$F_3^8(\alpha = 0.05) = 8.85$		
0	0	1.13	1.19	QME/QMEe = 1.36	$F_4^9(\alpha = 0.05) = 6.00$		
	SS	df	QM	QMLF/QMEe = 1.66	$F_4^5(\alpha = 0.05) = 6.26$		
Model	1.22	3	0.41	r^2 = 0.968	adjusted r^2 = 0.958		
Error	0.04	9	0.00				
Ee	0.01	4	0.00	Optimum A value = 147 rpm			
LF	0.03	5	0.01	Optimum GW = 2.47 g			
Total	1.26	12	0.10	Maximum [QA]c = 1.70 g/L			

Table S10. Results of the experimental design and analysis of the significance of the proposed model for lactic acid bacteria (LAB) counts at 24 h of fermentation. Y: response (log CFU/mL); Ŷ: expected response (log CFU/mL); NS: non-significant coefficient; SS: sum of squares; df: degrees of freedom; QM: quadratic means; M: model; E: total error; Ee: experimental error; LF: lack of fit; Var(Ee): variance of the error experimental. A: agitation speed; GW: kefir grains weight.

A	GW	Y	Ŷ	Coefficients	*t*	Model	
1	1	5.85	5.89	6.63	194.18	6.63	
1	-1	5.80	5.89	-0.31	10.71	-0.31	A
-1	1	6.51	6.50	-0.01	0.30	NS	GW
-1	-1	6.53	6.50	0.02	0.43	NS	A·GW
1.267	0	6.22	6.22	-0.06	0.84	NS	A^2
-1.267	0	6.86	6.99	-0.41	11.24	-0.41	GW^2
0	1.267	5.94	5.95	Mean response = 6.38			
0	-1.267	6.01	5.95	Central mean response = 6.63			
0	0	6.47	6.60	Var(Ee) = 0.02			
0	0	6.86	6.60	t (α < 0.05; df = 4) = 2.78			
0	0	6.63	6.60	QMM/QME = 61.71	$F_{10}^{2}(\alpha = 0.05) = 4.10$		
0	0	6.66	6.60	QMLF/QMM = 0.26	$F_{2}^{8}(\alpha = 0.05) = 19.37$		
0	0	6.54	6.60	QME/QMEe = 0.58	$F_{4}^{10}(\alpha = 0.05) = 5.96$		
	SS	df	QM	QMLF/QMEe = 0.29	$F_{4}^{6}(\alpha = 0.05) = 6.16$		
Model	1.52	2	0.76	r^2 = 0.925	adjusted r^2 = 0.910		
Error	0.12	10	0.01				
Ee	0.09	4	0.02	Optimum A value = 25 rpm			
LF	0.04	6	0.01	Optimum GW = 1.80 g			
Total	1.65	12	0.14	Maximum [LAB] = $10^{7.02}$ = 1.05×10^7 CFU/mL			

Table S11. Results of the experimental design and analysis of the significance of the proposed model for lactic acid bacteria (LAB) counts at 48 h of fermentation. Y: response (log CFU/mL); Ŷ: expected response (log CFU/mL); NS: non-significant coefficient; SS: sum of squares; df: degrees of freedom; QM: quadratic means; M: model; E: total error; Ee: experimental error; LF: lack of fit; Var(Ee): variance of the error experimental. A: agitation speed; GW: kefir grains weight.

A	GW	Y	Ŷ	Coefficients	*t*	Model	
1	1	5.12	5.16	7.05	94.78	7.05	
1	-1	5.06	5.16	-1.53	24.45	-1.53	A
-1	1	8.27	8.22	-0.01	0.20	NS	GW
-1	-1	8.31	8.22	0.02	0.28	NS	A·GW
1.267	0	5.66	5.23	-0.08	2.73	NS	A^2
-1.267	0	9.32	9.10	-0.47	6.40	-0.47	GW^2
0	1.267	6.36	6.40	Mean response = 6.90			
0	-1.267	6.46	6.40	Central mean response = 7.03			
0	0	7.12	7.16	Var(Ee) = 0.03			
0	0	6.80	7.16	t (α < 0.05; df = 4) = 2.78			
0	0	7.10	7.16	QMM/QME = 194.67	$F_{10}^{2}(\alpha = 0.05) = 4.10$		
0	0	6.93	7.16	QMLF/QMM = 0.26	$F_{2}^{8}(\alpha = 0.05) = 19.37$		
0	0	7.22	7.16	QME/QMEe = 1.64	$F_{4}^{10}(\alpha = 0.05) = 5.96$		
	SS	df	QM	QMLF/QMEe = 2.07	$F_{4}^{6}(\alpha = 0.05) = 6.16$		
Model	18.02	2	9.01	r^2 = 0.917	adjusted r^2 = 0.900		
Error	0.46	10	0.05				
Ee	0.11	4	0.03	Optimum A value = 25 rpm			
LF	0.35	6	0.06	Optimum GW = 1.80 g			
Total	18.48	12	1.54	Maximum [LAB] = $10^{8.99}$ = 9.77×10^{8} CFU/mL			

Table S12. Results of the experimental design and analysis of the significance of the proposed model for lactic acid bacteria (LAB) counts at 72 h of fermentation. Y: response (log CFU/mL); Ŷ: expected response (log CFU/mL); NS: non-significant coefficient; SS: sum of squares; df: degrees of freedom; QM: quadratic means; M: model; E: total error; Ee: experimental error; LF: lack of fit; Var(Ee): variance of the error experimental. A: agitation speed; GW: kefir grains weight.

A	GW	Y	Ŷ	Coefficients	t	Model	
1	1	4.28	4.60	6.55	194.18	6.55	
1	-1	4.77	4.60	-1.36	10.71	-1.36	A
-1	1	7.31	7.32	-0.16	0.30	NS	GW
-1	-1	7.40	7.32	-0.10	0.43	NS	A·GW
1.267	0	4.75	4.78	-0.08	0.84	NS	A^2
-1.267	0	8.04	8.23	-0.55	11.24	-0.55	GW^2
0	1.267	5.40	5.63	Mean response = 6.20			
0	-1.267	5.89	5.63	Central mean response = 6.55			
0	0	6.67	6.51	Var(Ee) = 0.04			
0	0	6.65	6.51	t ($\alpha < 0.05$; $df = 4$) = 2.78			
0	0	6.77	6.51	QMM/QME = 160.55	$F_{10}^{2}(\alpha = 0.05) = 4.10$		
0	0	6.33	6.51	QMLF/QMM = 0.26	$F_{2}^{8}(\alpha = 0.05) = 19.37$		
0	0	6.35	6.51	QME/QMEe = 1.13	$F_{4}^{10}(\alpha = 0.05) = 5.96$		
	SS	df	QM	QMLF/QMEe = 1.22	$F_{4}^{6}(\alpha = 0.05) = 6.16$		
Model	14.92	2	7.46	$r^2 = 0.970$	adjusted $r^2 = 0.964$		
Error	0.46	10	0.05				
Ee	0.16	4	0.04	Optimum A value = 25 rpm			
LF	0.30	6	0.05	Optimum GW = 1.80 g			
Total	15.38	12	1.28	Maximum [LAB] = $10^{8.28} = 1.91\times10^{8}$ CFU/mL			

Table S13. Results of the experimental design and analysis of the significance of the proposed model for acetic acid bacteria (AAB) counts at 24 h of fermentation. Y: response (log CFU/mL); Ŷ: expected response (log CFU/mL); NS: non-significant coefficient; SS: sum of squares; df: degrees of freedom; QM: quadratic means; M: model; E: total error; Ee: experimental error; LF: lack of fit; Var(Ee): variance of the error experimental. A: agitation speed; GW: kefir grains weight.

A	GW	Y	Ŷ	Coefficients	*t*	Model	
1	1	6.42	6.41	6.66	131.71	6.66	
1	-1	6.43	6.41	0.21	4.88	0.21	A
-1	1	5.92	5.99	0.02	0.18	NS	GW
-1	-1	5.88	5.99	-0.01	0.26	NS	A·GW
1.267	0	6.93	6.94	0.02	0.38	NS	A^2
-1.267	0	6.57	6.41	-0.48	9.48	-0.48	GW^2
0	1.267	5.99	5.91	Mean response = 6.41			
0	-1.267	5.92	5.91	Central mean response = 6.66			
0	0	6.65	6.67	Var(Ee) = 0.01			
0	0	6.83	6.67	t ($\alpha < 0.05$; $df = 4$) = 2.78			
0	0	6.62	6.67	QMM/QME = 71.44	$F_{10}^{2}(\alpha = 0.05) = 4.10$		
0	0	6.66	6.67	QMLF/QMM = 0.26	$F_{2}^{8}(\alpha = 0.05) = 19.37$		
0	0	6.52	6.67	QME/QMEe = 0.80	$F_{4}^{10}(\alpha = 0.05) = 5.96$		
	SS	df	QM	QMLF/QMEe = 0.66	$F_{4}^{6}(\alpha = 0.05) = 6.16$		
Model	1.48	2	0.74	$r^2 = 0.935$	adjusted $r^2 = 0.922$		
Error	0.10	10	0.01				
Ee	0.05	4	0.01	Optimum A value = 147 rpm			
LF	0.05	6	0.01	Optimum GW = 1.80 g			
Total	1.59	12	0.13	Maximum [AAB] = $10^{6.93} = 8.51\times10^{6}$ CFU/mL			

Table S14. Results of the experimental design and analysis of the significance of the proposed model for acetic acid bacteria (AAB) counts at 48 h of fermentation. Y: response (log CFU/mL); Ŷ: expected response (log CFU/mL); NS: non-significant coefficient; SS: sum of squares; df: degrees of freedom; QM: quadratic means; M: model; E: total error; Ee: experimental error; LF: lack of fit; Var(Ee): variance of the error experimental. A: agitation speed; GW: kefir grains weight.

A	GW	Y	Ŷ	Coefficients	*t*	Model	
1	1	5.46	5.45	5.57	102.50	5.57	
1	-1	5.48	5.45	0.24	5.34	0.24	A
-1	1	4.92	4.96	0.01	0.13	NS	GW
-1	-1	4.87	4.96	-0.02	0.27	NS	A·GW
1.267	0	5.75	5.86	-0.03	0.63	NS	A^2
-1.267	0	5.28	5.24	-0.35	6.43	-0.35	GW^2
0	1.267	5.04	4.99	Mean response = 5.36			
0	-1.267	4.99	4.99	Central mean response = 5.57			
0	0	5.43	5.55	Var(Ee) = 0.01			
0	0	5.76	5.55	t ($\alpha < 0.05$; $df = 4$) = 2.78			
0	0	5.57	5.55	QMM/QME = 58.98	$F_{10}^{2}(\alpha = 0.05) = 4.10$		
0	0	5.58	5.55	QMLF/QMM = 0.26	$F_{2}^{8}(\alpha = 0.05) = 19.37$		
0	0	5.49	5.55	QME/QMEe = 0.59	$F_{4}^{10}(\alpha = 0.05) = 5.96$		
	SS	df	QM	QMLF/QMEe = 0.32	$F_{4}^{6}(\alpha = 0.05) = 6.16$		
Model	1.05	2	0.52	$r^2 = 0.922$	adjusted $r^2 = 0.906$		
Error	0.09	10	0.01				
Ee	0.06	4	0.02	Optimum A value = 147 rpm			
LF	0.03	6	0.00	Optimum GW = 1.80 g			
Total	1.14	12	0.09	Maximum [AAB] = $10^{5.88} = 7.59\times10^{5}$ CFU/mL			

Table S15. Results of the experimental design and analysis of the significance of the proposed model for acetic acid bacteria (AAB) counts at 72 h of fermentation. Y: response (log CFU/mL); Ŷ: expected response (log CFU/mL); NS: non-significant coefficient; SS: sum of squares; df: degrees of freedom; QM: quadratic means; M: model; E: total error; Ee: experimental error; LF: lack of fit; Var(Ee): variance of the error experimental. A: agitation speed; GW: kefir grains weight.

A	GW	Y	Ŷ	Coefficients	*t*	Model	
1	1	4.88	4.87	5.00	87.18	5.00	
1	-1	4.90	4.87	0.22	4.52	0.22	A
-1	1	4.39	4.43	0.01	0.11	NS	GW
-1	-1	4.35	4.43	-0.01	0.23	NS	A·GW
1.267	0	5.14	5.25	-0.05	0.79	NS	A^2
-1.267	0	4.72	4.70	-0.33	5.70	-0.33	GW^2
0	1.267	4.51	4.45	Mean response = 4.80			
0	-1.267	4.45	4.45	Central mean response = 5.00			
0	0	4.85	4.98	Var(Ee) = 0.02			
0	0	5.14	4.98	t (α < 0.05; df = 4) = 2.78			
0	0	4.98	4.98	QMM/QME = 46.27		$F_{10}^{2}(\alpha = 0.05) = 4.10$	
0	0	5.13	4.98	QMLF/QMM = 0.26		$F_{2}^{8}(\alpha = 0.05) = 19.37$	
0	0	4.91	4.98	QME/QMEe = 0.57		$F_{4}^{10}(\alpha = 0.05) = 5.96$	
	SS	df	QM	QMLF/QMEe = 0.29		$F_{4}^{6}(\alpha = 0.05) = 6.16$	
Model	0.89	2	0.44	$r^2 = 0.902$		adjusted $r^2 = 0.883$	
Error	0.10	10	0.01				
Ee	0.07	4	0.02	Optimum A value = 147 rpm			
LF	0.03	6	0.00	Optimum GW = 1.80 g			
Total	0.98	12	0.08	Maximum [AAB] = $10^{5.28}$ = 1.91×10^5 CFU/mL			

Table S16. Results of the experimental design and analysis of the significance of the proposed model for yeast counts at 24 h of fermentation. Y: response (log CFU/mL); Ŷ: expected response (log CFU/mL); NS: non-significant coefficient; SS: sum of squares; df: degrees of freedom; QM: quadratic means; M: model; E: total error; Ee: experimental error; LF: lack of fit; Var(Ee): variance of the error experimental. A: agitation speed; GW: kefir grains weight.

A	GW	Y	Ŷ	Coefficients	*t*	Model	
1	1	6.64	6.58	6.80	112.72	6.80	
1	-1	6.66	6.58	0.24	4.64	0.24	A
-1	1	5.96	6.10	-0.01	0.17	NS	GW
-1	-1	5.92	6.10	-0.02	0.25	NS	A·GW
1.267	0	7.09	7.13	0.06	1.03	NS	A^2
-1.267	0	6.88	6.54	-0.49	8.22	-0.49	GW^2
0	1.267	6.06	6.04	Mean response = 6.56			
0	-1.267	6.13	6.04	Central mean response = 6.79			
0	0	6.66	6.83	Var(Ee) = 0.02			
0	0	7.00	6.83	t ($\alpha < 0.05$; $df = 4$) = 2.78			
0	0	6.77	6.83	QMM/QME = 29.25	$F_{10}^{2}(\alpha = 0.05) = 4.10$		
0	0	6.82	6.83	QMLF/QMM = 0.28	$F_{2}^{8}(\alpha = 0.05) = 19.37$		
0	0	6.67	6.83	QME/QMEe = 1.52	$F_{4}^{10}(\alpha = 0.05) = 5.96$		
	SS	df	QM	QMLF/QMEe = 1.87	$F_{4}^{6}(\alpha = 0.05) = 6.16$		
Model	1.65	2	0.83	r^2 = 0.854	adjusted r^2 = 0.825		
Error	0.28	10	0.03				
Ee	0.07	4	0.02	Optimum A value = 147 rpm			
LF	0.21	6	0.03	Optimum GW = 1.80 g			
Total	1.93	12	0.16	Maximum [Yeasts] = $10^{7.10}$ = 1.26×10^{7} CFU/mL			

Table S17. Results of the experimental design and analysis of the significance of the proposed model for yeast counts at 48 h of fermentation. Y: response (log CFU/mL); Ŷ: expected response (log CFU/mL); NS: non-significant coefficient; SS: sum of squares; df: degrees of freedom; QM: quadratic means; M: model; E: total error; Ee: experimental error; LF: lack of fit; Var(Ee): variance of the error experimental. A: agitation speed; GW: kefir grains weight.

A	GW	Y	Ŷ	Coefficients	*t*	Model	
1	1	6.90	6.89	7.02	101.66	7.02	
1	-1	6.92	6.89	0.32	5.57	0.32	A
-1	1	6.20	6.24	-0.01	0.16	NS	GW
-1	-1	6.15	6.24	0.02	0.23	NS	A·GW
1.267	0	7.27	7.40	-0.05	0.76	NS	A^2
-1.267	0	6.60	6.58	-0.42	6.18	-0.42	GW^2
0	1.267	6.30	6.31	Mean response = 6.75			
0	-1.267	6.37	6.31	Central mean response = 7.02			
0	0	6.86	6.99	Var(Ee) = 0.02			
0	0	7.27	6.99	t (α < 0.05; df = 4) = 2.78			
0	0	7.04	6.99	QMM/QME = 62.04		$F_{10}^{2}(\alpha = 0.05) = 4.10$	
0	0	6.98	6.99	QMLF/QMM = 0.26		$F_{2}^{8}(\alpha = 0.05) = 19.37$	
0	0	6.94	6.99	QME/QMEe = 0.56		$F_{4}^{10}(\alpha = 0.05) = 5.96$	
	SS	df	QM	QMLF/QMEe = 0.26		$F_{4}^{6}(\alpha = 0.05) = 6.16$	
Model	1.68	2	0.84	r^2 = 0.925		adjusted r^2 = 0.911	
Error	0.14	10	0.01				
Ee	0.10	4	0.02	Optimum A value = 147 rpm			
LF	0.04	6	0.01	Optimum GW = 1.80 g			
Total	1.82	12	0.15	Maximum [Yeasts] = $10^{7.43}$ = 2.69×10^{7} CFU/mL			

Table S18. Results of the experimental design and analysis of the significance of the proposed model for yeast counts at 72 h of fermentation. Y: response (log CFU/mL); Ŷ: expected response (log CFU/mL); NS: non-significant coefficient; SS: sum of squares; df: degrees of freedom; QM: quadratic means; M: model; E: total error; Ee: experimental error; LF: lack of fit; Var(Ee): variance of the error experimental. A: agitation speed; GW: kefir grains weight.

A	GW	Y	Ŷ	Coefficients	*t*	Model	
1	1	7.84	7.81	7.92	194.18	7.92	
1	-1	7.86	7.81	0.37	10.71	0.37	A
-1	1	7.03	7.08	-0.01	0.30	NS	GW
-1	-1	6.98	7.08	0.02	0.43	NS	A·GW
1.267	0	8.25	8.37	-0.03	0.84	NS	A^2
-1.267	0	7.49	7.44	-0.46	11.24	-0.46	GW^2
0	1.267	7.15	7.17	Mean response = 7.65			
0	-1.267	7.23	7.17	Central mean response = 7.92			
0	0	7.79	7.90	Var(Ee) = 0.01			
0	0	8.02	7.90	t (α < 0.05; df = 4) = 2.78			
0	0	7.99	7.90	QMM/QME = 143.73	$F_{10}^{2}(\alpha = 0.05) = 4.10$		
0	0	7.93	7.90	QMLF/QMM = 0.26	$F_{2}^{8}(\alpha = 0.05) = 19.37$		
0	0	7.87	7.90	QME/QMEe = 0.84	$F_{4}^{10}(\alpha = 0.05) = 5.96$		
	SS	df	QM	QMLF/QMEe = 0.73	$F_{4}^{6}(\alpha = 0.05) = 6.16$		
Model	2.04	2	1.02	$r^2 = 0.966$	adjusted $r^2 = 0.960$		
Error	0.07	10	0.01				
Ee	0.03	4	0.01	Optimum A value = 147 rpm			
LF	0.04	6	0.01	Optimum GW = 1.80 g			
Total	2.11	12	0.18	Maximum [Yeasts] = $10^{8.38} = 2.40\times10^{8}$ CFU/mL			

Table S19. Results of the experimental design and analysis of the significance of the proposed model for free biomass production ([X]p) at 24 h of fermentation. Y: response (g/L); Ŷ: expected response (g/L); NS: non-significant coefficient; SS: sum of squares; df: degrees of freedom; QM: quadratic means; M: model; E: total error; Ee: experimental error; LF: lack of fit; Var(Ee): variance of the error experimental. A: agitation speed; GW: kefir grains weight.

A	GW	Y	Ŷ	Coefficients	t	Model	
1	1	45.71	45.70	51.23	58.72	51.23	
1	-1	45.69	45.70	3.15	4.29	3.15	A
-1	1	39.81	39.41	0.82	1.12	NS	GW
-1	-1	39.64	39.41	-0.04	0.04	NS	A·GW
1.267	0	55.79	55.35	0.25	0.28	NS	A^2
-1.267	0	47.32	47.38	-8.81	10.16	-8.81	GW^2
0	1.267	39.28	37.22	Mean response = 46.48			
0	-1.267	34.76	37.22	Central mean response = 51.23			
0	0	51.66	51.36	Var(Ee) = 3.88			
0	0	50.12	51.36	t (α < 0.05; df = 4) = 2.78			
0	0	48.47	51.36	QMM/QME = 89.58	$F_{10}^{2}(\alpha = 0.05) = 4.10$		
0	0	53.46	51.36	QMLF/QMM = 0.26	$F_{2}^{8}(\alpha = 0.05) = 19.37$		
0	0	52.46	51.36	QME/QMEe = 0.68	$F_{4}^{10}(\alpha = 0.05) = 5.96$		
	SS	df	QM	QMLF/QMEe = 0.46	$F_{4}^{6}(\alpha = 0.05) = 6.16$		
Model	471.24	2	235.62	r^2 = 0.947	adjusted r^2 = 0.937		
Error	26.30	10	2.63				
Ee	15.51	4	3.88	Optimum A value = 147 rpm			
LF	10.80	6	1.80	Optimum GW = 1.80 g			
Total	497.55	12	41.46	Maximum [X]p = 55.21 g/L			

Table S20. Results of the experimental design and analysis of the significance of the proposed model for free biomass production ([X]p) at 48 h of fermentation. Y: response (g/L); Ŷ: expected response (g/L); NS: non-significant coefficient; SS: sum of squares; df: degrees of freedom; QM: quadratic means; M: model; E: total error; Ee: experimental error; LF: lack of fit; Var(Ee): variance of the error experimental. A: agitation speed; GW: kefir grains weight.

A	GW	Y	Ŷ	Coefficients	*t*	Model	
1	1	46.15	46.12	51.18	84.67	51.18	
1	-1	46.97	46.12	3.20	6.29	3.20	A
-1	1	40.56	39.73	1.15	2.27	NS	GW
-1	-1	40.18	39.73	-0.30	0.44	NS	A·GW
1.267	0	56.91	55.79	1.01	1.68	NS	A^2
-1.267	0	48.47	47.69	-8.81	14.67	-8.81	GW^2
0	1.267	40.37	37.59	Mean response = 46.85			
0	-1.267	33.46	37.59	Central mean response = 51.20			
0	0	52.22	51.74	Var(Ee) = 1.86			
0	0	52.31	51.74	t (α < 0.05; df = 4) = 2.78			
0	0	50.07	51.74	QMM/QME = 63.74	$F_{10}^{2}(\alpha = 0.05) = 4.10$		
0	0	51.99	51.74	QMLF/QMM = 0.27	$F_{2}^{8}(\alpha = 0.05) = 19.37$		
0	0	49.39	51.74	QME/QMEe = 2.00	$F_{4}^{10}(\alpha = 0.05) = 5.96$		
	SS	df	QM	QMLF/QMEe = 2.66	$F_{4}^{6}(\alpha = 0.05) = 6.16$		
Model	474.16	2	237.08	r^2 = 0.927	adjusted r^2 = 0.913		
Error	37.19	10	3.72				
Ee	7.44	4	1.86	Optimum A value = 147 rpm			
LF	29.75	6	4.96	Optimum GW = 1.80 g			
Total	511.34	12	42.61	Maximum [X]p = 55.23 g/L			

Table S21. Results of the experimental design and analysis of the significance of the proposed model for free biomass production ([X]p) at 72 h of fermentation. Y: response (g/L); Ŷ: expected response (g/L); NS: non-significant coefficient; SS: sum of squares; df: degrees of freedom; QM: quadratic means; M: model; E: total error; Ee: experimental error; LF: lack of fit; Var(Ee): variance of the error experimental. A: agitation speed; GW: kefir grains weight.

A	GW	Y	Ŷ	Coefficients	*t*	Model	
1	1	49.73	49.80	50.52	45.08	50.52	
1	-1	49.89	49.80	2.55	9.19	2.55	A
-1	1	44.64	44.70	-0.07	3.25	NS	GW
-1	-1	44.29	44.70	-0.13	0.36	NS	A·GW
1.267	0	52.34	53.40	-0.63	2.84	NS	A^2
-1.267	0	46.26	46.94	-2.92	17.40	-2.92	GW^2
0	1.267	45.37	45.49	Mean response = 48.55			
0	-1.267	45.89	45.49	Central mean response = 50.56			
0	0	49.43	50.17	Var(Ee) = 1.26			
0	0	52.38	50.17	t (α < 0.05; df = 4) = 2.78			
0	0	50.71	50.17	QMM/QME = 58.79	$F_{10}^{2}(\alpha = 0.05) = 4.10$		
0	0	50.30	50.17	QMLF/QMM = 0.26	$F_{2}^{8}(\alpha = 0.05) = 19.37$		
0	0	49.96	50.17	QME/QMEe = 0.61	$F_{4}^{10}(\alpha = 0.05) = 5.96$		
	SS	df	QM	QMLF/QMEe = 0.36	$F_{4}^{6}(\alpha = 0.05) = 6.16$		
Model	90.83	2	45.42	r^2 = 0.922	adjusted r^2 = 0.906		
Error	7.73	10	0.77				
Ee	5.04	4	1.26	Optimum A value = 147 rpm			
LF	2.69	6	0.45	Optimum GW = 1.80 g			
Total	98.56	12	8.21	Maximum [X]p = 53.76 g/L			

Table S22. Results of the experimental design and analysis of the significance of the proposed model for lactic acid production ([LA]p) at 24 h of fermentation. Y: response (g/L); Ŷ: expected response (g/L); NS: non-significant coefficient; SS: sum of squares; df: degrees of freedom; QM: quadratic means; M: model; E: total error; Ee: experimental error; LF: lack of fit; Var(Ee): variance of the error experimental. A: agitation speed; GW: kefir grains weight.

A	GW	Y	Ŷ	Coefficients	*t*	Model	
1	1	1.31	1.21	0.93	29.73	0.93	
1	-1	0.86	0.91	0.23	8.77	0.23	A
-1	1	0.78	0.75	0.15	5.66	0.15	GW
-1	-1	0.49	0.45	0.04	1.12	NS	A·GW
1.267	0	1.02	1.04	-0.13	4.18	-0.13	A^2
-1.267	0	0.41	0.46	0.06	1.93	NS	GW^2
0	1.267	1.15	1.15	Mean response = 0.89			
0	-1.267	0.89	0.77	Central mean response = 0.93			
0	0	0.84	0.96	Var(Ee) = 0.005			
0	0	1.04	0.96	t (α < 0.05; df = 4) = 2.78			
0	0	0.90	0.96	QMM/QME = 33.45		$F_9^3(\alpha = 0.05) = 3.86$	
0	0	0.93	0.96	QMLF/QMM = 0.40		$F_3^8(\alpha = 0.05) = 8.85$	
0	0	0.93	0.96	QME/QMEe = 1.26		$F_4^9(\alpha = 0.05) = 6.00$	
	SS	df	QM	QMLF/QMEe = 1.47		$F_4^5(\alpha = 0.05) = 6.62$	
Model	0.626	3	0.209	r^2 = 0.918		adjusted r^2 = 0.890	
Error	0.056	9	0.006				
Ee	0.020	4	0.005	Optimum A value = 128 rpm			
LF	0.036	5	0.007	Optimum GW = 2.81 g			
Total	0.682	12	0.057	Maximum [LA]p = 1.22 g/L			

Table S23. Results of the experimental design and analysis of the significance of the proposed model for lactic acid production ([LA]p) at 48 h of fermentation. Y: response (g/L); Ŷ: expected response (g/L); NS: non-significant coefficient; SS: sum of squares; df: degrees of freedom; QM: quadratic means; M: model; E: total error; Ee: experimental error; LF: lack of fit; Var(Ee): variance of the error experimental. A: agitation speed; GW: kefir grains weight.

A	GW	Y	Ŷ	Coefficients	*t*	Model	
1	1	0.65	0.64	0.43	24.02	0.43	
1	-1	0.33	0.31	0.11	7.57	0.11	A
-1	1	0.30	0.28	0.10	6.80	0.10	GW
-1	-1	0.22	0.20	0.06	3.09	0.06	A·GW
1.267	0	0.42	0.45	-0.10	5.39	-0.10	A^2
-1.267	0	0.14	0.16	0.04	2.34	NS	GW^2
0	1.267	0.64	0.59	Mean response = 0.40			
0	-1.267	0.37	0.33	Central mean response = 0.43			
0	0	0.40	0.46	Var(Ee) = 0.002			
0	0	0.48	0.46	t (α < 0.05; df = 4) = 2.78			
0	0	0.44	0.46	QMM/QME = 29.75	$F_9^3(\alpha = 0.05) = 3.86$		
0	0	0.38	0.46	QMLF/QMM = 0.52	$F_3^8(\alpha = 0.05) = 8.85$		
0	0	0.46	0.46	QME/QMEe = 1.19	$F_4^9(\alpha = 0.05) = 6.00$		
	SS	df	QM	QMLF/QMEe = 1.39	$F_4^5(\alpha = 0.05) = 6.62$		
Model	0.234	4	0.059	r^2 = 0.937	adjusted r^2 = 0.905		
Error	0.016	8	0.002				
Ee	0.007	4	0.002	Optimum A value = 134 rpm			
LF	0.009	4	0.002	Optimum GW = 2.81 g			
Total	0.250	12	0.021	Maximum [LA]p = 0.66 g/L			

Table S24. Results of the experimental design and analysis of the significance of the proposed model for lactic acid production ([LA]p) at 72 h of fermentation. Y: response (g/L); Ŷ: expected response (g/L); NS: non-significant coefficient; SS: sum of squares; df: degrees of freedom; QM: quadratic means; M: model; E: total error; Ee: experimental error; LF: lack of fit; Var(Ee): variance of the error experimental. A: agitation speed; GW: kefir grains weight.

A	GW	Y	Ŷ	Coefficients	*t*	Model	
1	1	0.62	0.58	0.40	24.02	0.40	
1	-1	0.34	0.32	0.11	7.57	0.11	A
-1	1	0.26	0.23	0.06	6.80	0.06	GW
-1	-1	0.25	0.24	0.07	3.09	0.07	A·GW
1.267	0	0.38	0.43	-0.08	5.39	-0.08	A^2
-1.267	0	0.14	0.16	0.03	2.34	NS	GW^2
0	1.267	0.51	0.50	Mean response = 0.38			
0	-1.267	0.36	0.34	Central mean response = 0.41			
0	0	0.43	0.42	Var(Ee) = 0.001			
0	0	0.36	0.42	t ($\alpha < 0.05$; $df = 4$) = 2.78			
0	0	0.39	0.42	QMM/QME = 26.67	$F_8^4(\alpha = 0.05) = 3.84$		
0	0	0.41	0.42	QMLF/QMM = 0.52	$F_4^8(\alpha = 0.05) = 6.04$		
0	0	0.44	0.42	QME/QMEe = 1.33	$F_4^8(\alpha = 0.05) = 6.04$		
	SS	df	QM	QMLF/QMEe = 1.66	$F_4^4(\alpha = 0.05) = 6.39$		
Model	0.160	4	0.040	r^2 = 0.930	adjusted r^2 = 0.895		
Error	0.012	8	0.001				
Ee	0.005	4	0.001	Optimum A value = 143 rpm			
LF	0.007	4	0.002	Optimum GW = 2.81 g			
Total	0.172	12	0.014	Maximum [LA]p = 0.60 g/L			

Table S25. Results of the experimental design and analysis of the significance of the proposed model for acetic acid production ([AA]p) at 24 h of fermentation. Y: response (g/L); Ŷ: expected response (g/L); NS: non-significant coefficient; SS: sum of squares; df: degrees of freedom; QM: quadratic means; M: model; E: total error; Ee: experimental error; LF: lack of fit; Var(Ee): variance of the error experimental. A: agitation speed; GW: kefir grains weight.

A	GW	Y	Ŷ	Coefficients	*t*	Model	
1	1	0.22	0.23	0.15	28.68	0.15	
1	-1	0.09	0.11	0.03	7.59	0.03	A
-1	1	0.12	0.11	0.03	7.76	0.03	GW
-1	-1	0.09	0.09	0.03	4.26	0.03	A·GW
1.267	0	0.19	0.17	-0.02	3.14	-0.02	A^2
-1.267	0	0.07	0.08	-0.07	1.33	NS	GW^2
0	1.267	0.18	0.20	Mean response = 0.14			
0	-1.267	0.11	0.11	Central mean response = 0.16			
0	0	0.14	0.15	Var(Ee) = 0.0001			
0	0	0.17	0.15	t (α < 0.05; df = 4) = 2.78			
0	0	0.16	0.15	QMM/QME = 18.76	$F_8^4(\alpha = 0.05) = 3.84$		
0	0	0.14	0.15	QMLF/QMM = 0.54	$F_4^8(\alpha = 0.05) = 6.04$		
0	0	0.17	0.15	QME/QMEe = 1.94	$F_4^8(\alpha = 0.05) = 6.04$		
	SS	df	QM	QMLF/QMEe = 2.88	$F_4^4(\alpha = 0.05) = 6.39$		
Model	0.022	4	0.005	r^2 = 0.930	adjusted r^2 = 0.895		
Error	0.002	8	0.000				
Ee	0.001	4	0.000	Optimum A value = 147 rpm			
LF	0.002	4	0.000	Optimum GW = 2.81 g			
Total	0.024	12	0.002	Maximum [AA]p = 0.26 g/L			

Table S26. Results of the experimental design and analysis of the significance of the proposed model for acetic acid production ([AA]p) at 48 h of fermentation. Y: response (g/L); $\hat{Y}$: expected response (g/L); NS: non-significant coefficient; SS: sum of squares; df: degrees of freedom; QM: quadratic means; M: model; E: total error; Ee: experimental error; LF: lack of fit; Var(Ee): variance of the error experimental. A: agitation speed; GW: kefir grains weight.

A	GW	Y	$\hat{Y}$	Coefficients	*t*	Model	
1	1	0.44	0.44	0.31	41.50	0.31	
1	-1	0.25	0.27	0.07	11.55	0.07	A
-1	1	0.35	0.30	0.09	13.93	0.09	GW
-1	-1	0.15	0.13	-0.00	0.30	NS	A·GW
1.267	0	0.38	0.36	-0.02	3.34	-0.02	A^2
-1.267	0	0.13	0.18	0.01	1.02	NS	GW^2
0	1.267	0.40	0.42	Mean response = 0.30			
0	-1.267	0.21	0.20	Central mean response = 0.31			
0	0	0.32	0.31	Var(Ee) = 0.0003			
0	0	0.30	0.31	t ($\alpha < 0.05$; $df = 4$) = 2.78			
0	0	0.30	0.31	QMM/QME = 38.86	$F_9^3(\alpha = 0.05) = 3.86$		
0	0	0.33	0.31	QMLF/QMM = 0.40	$F_3^8(\alpha = 0.05) = 8.85$		
0	0	0.28	0.31	QME/QMEe = 2.90	$F_4^9(\alpha = 0.05) = 6.00$		
	SS	df	QM	QMLF/QMEe = 4.43	$F_4^5(\alpha = 0.05) = 6.62$		
Model	0.093	3	0.031	$r^2 = 0.928$	adjusted $r^2 = 0.904$		
Error	0.007	9	0.001				
Ee	0.001	4	0.000	Optimum A value = 147 rpm			
LF	0.006	5	0.001	Optimum GW = 2.81 g			
Total	0.101	12	0.008	Maximum [AA]p = 0.47 g/L			

Table S27. Results of the experimental design and analysis of the significance of the proposed model for acetic acid production ([AA]p) at 72 h of fermentation. Y: response (g/L); Ŷ: expected response (g/L); NS: non-significant coefficient; SS: sum of squares; df: degrees of freedom; QM: quadratic means; M: model; E: total error; Ee: experimental error; LF: lack of fit; Var(Ee): variance of the error experimental. A: agitation speed; GW: kefir grains weight.

A	GW	Y	Ŷ	Coefficients	*t*	Model	
1	1	0.69	0.66	0.37	26.60	0.37	
1	-1	0.22	0.24	0.12	10.05	0.12	A
-1	1	0.44	0.43	0.21	18.17	0.21	GW
-1	-1	0.00	0.00	0.01	0.48	NS	A·GW
1.267	0	0.45	0.45	-0.04	3.27	-0.04	A^2
-1.267	0	0.15	0.16	0.01	0.95	NS	GW^2
0	1.267	0.64	0.65	Mean response = 0.35			
0	-1.267	0.15	0.11	Central mean response = 0.37			
0	0	0.41	0.38	Var(Ee) = 0.001			
0	0	0.32	0.38	t ($\alpha < 0.05$; $df = 4$) = 2.78			
0	0	0.38	0.38	QMM/QME = 182.25		$F_9^3(\alpha = 0.05) = 3.86$	
0	0	0.37	0.38	QMLF/QMM = 0.38		$F_3^8(\alpha = 0.05) = 8.85$	
0	0	0.37	0.38	QME/QMEe = 0.81		$F_4^9(\alpha = 0.05) = 6.00$	
	SS	df	QM	QMLF/QMEe = 0.65		$F_4^5(\alpha = 0.05) = 6.62$	
Model	0.110	3	0.037	$r^2 = 0.984$		adjusted $r^2 = 0.978$	
Error	0.004	9	0.000				
Ee	0.003	4	0.001	Optimum A value = 147 rpm			
LF	0.001	5	0.000	Optimum GW = 2.81 g			
Total	0.115	12	0.010	Maximum [AA]p = 0.72 g/L			

Table S28. Results of the experimental design and analysis of the significance of the proposed model for ethanol production ([EtOH]p) at 24 h of fermentation. Y: response (g/L); Ŷ: expected response (g/L); NS: non-significant coefficient; SS: sum of squares; df: degrees of freedom; QM: quadratic means; M: model; E: total error; Ee: experimental error; LF: lack of fit; Var(Ee): variance of the error experimental. A: agitation speed; GW: kefir grains weight.

A	GW	Y	Ŷ	Coefficients	*t*	Model	
1	1	0.99	0.99	1.01	46.37	1.01	
1	-1	0.66	0.62	-0.11	6.12	-0.11	A
-1	1	1.22	1.21	0.18	9.87	0.18	GW
-1	-1	0.96	0.85	0.02	0.64	NS	A·GW
1.267	0	0.70	0.72	-0.10	4.56	-0.10	A^2
-1.267	0	0.92	1.00	0.01	0.26	NS	GW^2
0	1.267	1.26	1.25	Mean response = 0.96			
0	-1.267	0.69	0.79	Central mean response = 1.02			
0	0	1.01	1.02	Var(Ee) = 0.002			
0	0	1.02	1.02	t ($\alpha < 0.05$; $df = 4$) = 2.78			
0	0	0.96	1.02	QMM/QME = 27.60		$F_9^3(\alpha = 0.05) = 3.86$	
0	0	1.02	1.02	QMLF/QMM = 0.41		$F_3^8(\alpha = 0.05) = 8.85$	
0	0	1.10	1.02	QME/QMEe = 1.88		$F_4^9(\alpha = 0.05) = 6.00$	
	SS	df	QM	QMLF/QMEe = 2.58		$F_4^5(\alpha = 0.05) = 6.62$	
Model	0.379	3	0.126	$r^2 = 0.902$		adjusted $r^2 = 0.869$	
Error	0.041	9	0.005				
Ee	0.010	4	0.002	Optimum A value = 61 rpm			
LF	0.031	5	0.006	Optimum GW = 2.81 g			
Total	0.420	12	0.035	Maximum [EtOH]p = 1.27 g/L			

Table S29. Results of the experimental design and analysis of the significance of the proposed model for ethanol production ([EtOH]p) at 48 h of fermentation. Y: response (g/L); Ŷ: expected response (g/L); NS: non-significant coefficient; SS: sum of squares; df: degrees of freedom; QM: quadratic means; M: model; E: total error; Ee: experimental error; LF: lack of fit; Var(Ee): variance of the error experimental. A: agitation speed; GW: kefir grains weight.

A	GW	Y	Ŷ	Coefficients	*t*	Model	
1	1	0.99	1.02	1.45	50.29	1.45	
1	-1	0.35	0.25	-0.33	13.70	-0.33	A
-1	1	1.82	1.96	0.52	21.43	0.52	GW
-1	-1	0.65	0.65	-0.13	4.09	-0.13	A·GW
1.267	0	0.46	0.52	-0.32	11.12	-0.32	A^2
-1.267	0	1.46	1.36	-0.16	5.69	-0.16	GW^2
0	1.267	1.98	1.85	Mean response = 1.19			
0	-1.267	0.44	0.53	Central mean response = 1.45			
0	0	1.44	1.45	Var(Ee) = 0.004			
0	0	1.54	1.45	t (α < 0.05; df = 4) = 2.78			
0	0	1.35	1.45	QMM/QME = 56.97		$F_7^5(\alpha = 0.05) = 3.97$	
0	0	1.46	1.45	QMLF/QMM = 0.64		$F_5^8(\alpha = 0.05) = 4.82$	
0	0	1.46	1.45	QME/QMEe = 2.88		$F_4^7(\alpha = 0.05) = 6.09$	
	SS	df	QM	QMLF/QMEe = 5.38		$F_4^3(\alpha = 0.05) = 6.59$	
Model	3.49	5	0.70	r^2 = 0.976		adjusted r^2 = 0.959	
Error	0.09	7	0.01				
Ee	0.02	4	0.00	Optimum A value = 50 rpm			
LF	0.07	3	0.02	Optimum GW = 2.81 g			
Total	3.57	12	0.30	Maximum [EtOH]p = 2.04 g/L			

Table S30. Results of the experimental design and analysis of the significance of the proposed model for ethanol production ([EtOH]p) at 72 h of fermentation. Y: response (g/L); Ŷ: expected response (g/L); NS: non-significant coefficient; SS: sum of squares; df: degrees of freedom; QM: quadratic means; M: model; E: total error; Ee: experimental error; LF: lack of fit; Var(Ee): variance of the error experimental. A: agitation speed; GW: kefir grains weight.

A	GW	Y	Ŷ	Coefficients	*t*	Model	
1	1	2.32	2.42	2.92	24.04	2.92	
1	-1	0.36	0.27	-0.59	5.74	-0.59	A
-1	1	3.50	3.59	1.07	10.53	1.07	GW
-1	-1	2.11	1.44	0.14	1.05	NS	A·GW
1.267	0	0.65	0.59	-0.99	8.23	-0.99	A^2
-1.267	0	1.66	2.07	0.02	0.13	NS	GW^2
0	1.267	4.50	4.29	Mean response = 2.37			
0	-1.267	1.04	1.57	Central mean response = 2.94			
0	0	2.72	2.93	Var(Ee) = 0.07			
0	0	2.58	2.93	t ($\alpha < 0.05$; $df = 4$) = 2.78			
0	0	3.09	2.93	QMM/QME = 37.45	$F_9^3(\alpha = 0.05) = 3.86$		
0	0	3.16	2.93	QMLF/QMM = 0.40	$F_3^8(\alpha = 0.05) = 8.85$		
0	0	3.16	2.93	QME/QMEe = 1.88	$F_4^9(\alpha = 0.05) = 6.00$		
	SS	df	QM	QMLF/QMEe = 2.59	$F_4^5(\alpha = 0.05) = 6.62$		
Model	15.85	3	5.28	$r^2 = 0.926$	adjusted $r^2 = 0.901$		
Error	1.27	9	0.14				
Ee	0.30	4	0.07	Optimum A value = 76 rpm			
LF	0.97	5	0.19	Optimum GW = 2.81 g			
Total	17.12	12	1.43	Maximum [EtOH]p = 4.36 g/L			

Table S31. Results of the experimental design and analysis of the significance of the proposed model for glycerol production ([GOH]p) at 24 h of fermentation. Y: response (g/L); Ŷ: expected response (g/L); NS: non-significant coefficient; SS: sum of squares; df: degrees of freedom; QM: quadratic means; M: model; E: total error; Ee: experimental error; LF: lack of fit; Var(Ee): variance of the error experimental. A: agitation speed; GW: kefir grains weight.

A	GW	Y	Ŷ	Coefficients	*t*	Model	
1	1	0.200	0.199	0.20	112.59	0.20	
1	-1	0.200	0.199	0.01	5.79	0.01	A
-1	1	0.179	0.181	0.00	0.17	NS	GW
-1	-1	0.178	0.181	-0.00	0.25	NS	A·GW
1.267	0	0.213	0.215	0.00	0.10	NS	A^2
-1.267	0	0.197	0.193	-0.01	7.63	-0.01	GW^2
0	1.267	0.182	0.182	Mean response = 0.196			
0	-1.267	0.184	0.182	Central mean response = 0.204			
0	0	0.200	0.204	Var(Ee) = 0.0000			
0	0	0.210	0.204	t (α < 0.05; df = 4) = 2.78			
0	0	0.204	0.204	QMM/QME = 68.64	$F_{10}^{2}(\alpha = 0.05) = 4.10$		
0	0	0.205	0.204	QMLF/QMM = 0.26	$F_{2}^{8}(\alpha = 0.05) = 19.37$		
0	0	0.201	0.204	QME/QMEe = 0.67	$F_{4}^{10}(\alpha = 0.05) = 5.96$		
	SS	df	QM	QMLF/QMEe = 0.45	$F_{4}^{6}(\alpha = 0.05) = 6.16$		
Model	0.0015	2	0.0008	r^2 = 0.932	adjusted r^2 = 0.919		
Error	0.0001	10	0.0000				
Ee	0.0001	4	0.0000	Optimum A value = 147 rpm			
LF	0.0000	6	0.0000	Optimum GW = 1.80 g			
Total	0.0016	12	0.0001	Maximum [GOH]p = 0.220 g/L			

Table S32. Results of the experimental design and analysis of the significance of the proposed model for glycerol production ([GOH]p) at 48 h of fermentation. Y: response (g/L); Ŷ: expected response (g/L); NS: non-significant coefficient; SS: sum of squares; df: degrees of freedom; QM: quadratic means; M: model; E: total error; Ee: experimental error; LF: lack of fit; Var(Ee): variance of the error experimental. A: agitation speed; GW: kefir grains weight.

A	GW	Y	Ŷ	Coefficients	*t*	Model	
1	1	1.271	1.267	1.29	98.86	1.29	
1	-1	1.271	1.267	0.06	5.38	0.06	A
-1	1	1.141	1.149	0.00	0.10	NS	GW
-1	-1	1.132	1.149	0.00	0.15	NS	A·GW
1.267	0	1.338	1.360	0.00	0.65	NS	A^2
-1.267	0	1.215	1.210	-0.08	5.93	-0.08	GW^2
0	1.267	1.160	1.161	Mean response = 1.242			
0	-1.267	1.173	1.161	Central mean response = 1.290			
0	0	1.263	1.285	Var(Ee) = 0.0009			
0	0	1.339	1.285	t ($\alpha < 0.05$; $df = 4$) = 2.78			
0	0	1.290	1.285	QMM/QME = 60.66	$F_{10}^{2}(\alpha = 0.05) = 4.10$		
0	0	1.283	1.285	QMLF/QMM = 0.26	$F_{2}^{8}(\alpha = 0.05) = 19.37$		
0	0	1.272	1.285	QME/QMEe = 0.53	$F_{4}^{10}(\alpha = 0.05) = 5.96$		
	SS	df	QM	QMLF/QMEe = 0.21	$F_{4}^{6}(\alpha = 0.05) = 6.16$		
Model	0.0556	2	0.0278	$r^2 = 0.971$	adjusted $r^2 = 0.961$		
Error	0.0046	10	0.0005				
Ee	0.0035	4	0.0009	Optimum A value = 147 rpm			
LF	0.0011	6	0.0002	Optimum GW = 1.80 g			
Total	1.6300	12	0.0050	Maximum [GOH]p = 1.359 g/L			

Table S33. Results of the experimental design and analysis of the significance of the proposed model for glycerol production ([GOH]p) at 72 h of fermentation. Y: response (g/L); Ŷ: expected response (g/L); NS: non-significant coefficient; SS: sum of squares; df: degrees of freedom; QM: quadratic means; M: model; E: total error; Ee: experimental error; LF: lack of fit; Var(Ee): variance of the error experimental. A: agitation speed; GW: kefir grains weight.

A	GW	Y	Ŷ	Coefficients	*t*	Model	
1	1	1.595	1.591	1.62	158.36	1.62	
1	-1	1.600	1.591	0.08	8.70	0.08	A
-1	1	1.432	1.442	0.00	0.24	NS	GW
-1	-1	1.421	1.442	0.00	0.35	NS	A·GW
1.267	0	1.679	1.707	0.00	1.01	NS	A^2
-1.267	0	1.524	1.518	-0.10	9.47	-0.10	GW^2
0	1.267	1.455	1.458	Mean response = 1.559			
0	-1.267	1.472	1.458	Central mean response = 1.618			
0	0	1.585	1.613	Var(Ee) = 0.0005			
0	0	1.632	1.613	t ($\alpha < 0.05$; $df = 4$) = 2.78			
0	0	1.637	1.613	QMM/QME = 110.85	$F_{10}^{2}(\alpha = 0.05) = 4.10$		
0	0	1.635	1.613	QMLF/QMM = 0.26	$F_{2}^{8}(\alpha = 0.05) = 19.37$		
0	0	1.603	1.613	QME/QMEe = 0.75	$F_{4}^{10}(\alpha = 0.05) = 5.96$		
	SS	df	QM	QMLF/QMEe = 0.58	$F_{4}^{6}(\alpha = 0.05) = 6.16$		
Model	0.0880	2	0.0440	$r^2 = 0.957$	adjusted $r^2 = 0.948$		
Error	0.0040	10	0.0004				
Ee	0.0021	4	0.0005	Optimum A value = 147 rpm			
LF	0.0018	6	0.0003	Optimum GW = 1.80 g			
Total	2.8801	12	0.0077	Maximum [GOH]p = 1.711 g/L			

Table S34. Results of the experimental design and analysis of the significance of the proposed model for antibacterial activity production ([AAct]p) at 24 h of fermentation. Y: response (g/L); Ŷ: expected response (g/L); NS: non-significant coefficient; SS: sum of squares; df: degrees of freedom; QM: quadratic means; M: model; E: total error; Ee: experimental error; LF: lack of fit; Var(Ee): variance of the error experimental. A: agitation speed; GW: kefir grains weight.

A	GW	Y	Ŷ	Coefficients	*t*	Model	
1	1	20.15	20.04	18.12	65.20	18.12	
1	-1	16.05	16.19	1.54	6.60	1.54	A
-1	1	17.23	16.95	1.92	8.23	1.92	GW
-1	-1	13.35	13.11	0.06	0.18	NS	A·GW
1.267	0	18.89	18.83	-0.78	2.81	-0.78	A^2
-1.267	0	14.55	14.92	-0.77	2.80	-0.77	GW^2
0	1.267	19.05	19.32	Mean response = 17.26			
0	-1.267	14.40	14.44	Central mean response = 18.15			
0	0	18.25	18.12	Var(Ee) = 0.39			
0	0	18.16	18.12	t ($\alpha < 0.05$; $df = 4$) = 2.78			
0	0	18.07	18.12	QMM/QME = 50.95	$F_8^4(\alpha = 0.05) = 3.84$		
0	0	17.25	18.12	QMLF/QMM = 0.50	$F_4^8(\alpha = 0.05) = 6.04$		
0	0	19.01	18.12	QME/QMEe = 0.62	$F_4^8(\alpha = 0.05) = 6.04$		
	SS	df	QM	QMLF/QMEe = 0.25	$F_4^4(\alpha = 0.05) = 6.39$		
Model	50.00	4	12.50	$r^2 = 0.962$	adjusted $r^2 = 0.943$		
Error	1.96	8	0.25				
Ee	1.57	4	0.39	Optimum A value = 134 rpm			
LF	0.39	4	0.10	Optimum GW = 2.79 g			
Total	51.96	12	4.33	Maximum [AAct]p = 20.02 g/L			

Table S35. Results of the experimental design and analysis of the significance of the proposed model for antibacterial activity production ([AAct]p) at 48 h of fermentation. Y: response (g/L); Ŷ: expected response (g/L); NS: non-significant coefficient; SS: sum of squares; df: degrees of freedom; QM: quadratic means; M: model; E: total error; Ee: experimental error; LF: lack of fit; Var(Ee): variance of the error experimental. A: agitation speed; GW: kefir grains weight.

A	GW	Y	Ŷ	Coefficients	*t*	Model	
1	1	21.34	21.09	18.85	64.84	18.85	
1	-1	16.79	16.17	2.85	11.64	2.85	A
-1	1	18.03	17.31	3.42	13.98	3.42	GW
-1	-1	9.65	8.56	-0.96	2.91	-0.96	A·GW
1.267	0	19.76	20.23	-1.39	4.79	-1.39	A^2
-1.267	0	11.82	13.02	-1.69	5.83	-1.69	GW^2
0	1.267	19.93	20.47	Mean response = 17.15			
0	-1.267	10.69	11.82	Central mean response = 18.99			
0	0	19.09	18.85	Var(Ee) = 0.43			
0	0	19.00	18.85	t (α < 0.05; df = 4) = 2.78			
0	0	18.90	18.85	QMM/QME = 33.12	$F_7^5(\alpha = 0.05) = 3.97$		
0	0	18.04	18.85	QMLF/QMM = 0.64	$F_5^8(\alpha = 0.05) = 4.82$		
0	0	19.89	18.85	QME/QMEe = 2.39	$F_4^7(\alpha = 0.05) = 6.09$		
	SS	df	QM	QMLF/QMEe = 4.25	$F_4^3(\alpha = 0.05) = 6.59$		
Model	170.72	5	34.14	r^2 = 0.959	adjusted r^2 = 0.930		
Error	7.22	7	1.03				
Ee	1.72	4	0.43	Optimum A value = 122 rpm			
LF	5.49	3	1.83	Optimum GW = 2.44 g			
Total	177.94	12	14.83	Maximum [AAct]p = 20.92 g/L			

Table S36. Results of the experimental design and analysis of the significance of the proposed model for antibacterial activity production ([AAct]p) at 72 h of fermentation. Y: response (g/L); Ŷ: expected response (g/L); NS: non-significant coefficient; SS: sum of squares; df: degrees of freedom; QM: quadratic means; M: model; E: total error; Ee: experimental error; LF: lack of fit; Var(Ee): variance of the error experimental, A: agitation speed; GW: kefir grains weight.

A	GW	Y	Ŷ	Coefficients	*t*	Model	
1	1	47.47	48.89	41.91	64.79	41.91	
1	-1	37.35	34.39	6.38	11.73	6.38	A
-1	1	40.10	36.14	7.25	13.33	7.25	GW
-1	-1	22.68	21.64	-1.82	2.50	NS	A·GW
1.267	0	43.96	44.63	-3.34	5.19	-3.34	A^2
-1.267	0	25.07	28.47	-3.30	5.14	-3.30	GW^2
0	1.267	44.33	45.79	Mean response = 38.22			
0	-1.267	24.81	27.42	Central mean response = 42.33			
0	0	42.47	41.91	Var(Ee) = 2.13			
0	0	42.26	41.91	t ($\alpha < 0.05$; $df = 4$) = 2.78			
0	0	42.05	41.91	QMM/QME = 33.12		$F_8^4(\alpha = 0.05) = 3.84$	
0	0	40.13	41.91	QMLF/QMM = 0.64		$F_4^8(\alpha = 0.05) = 6.04$	
0	0	44.24	41.91	QME/QMEe = 2.39		$F_4^8(\alpha = 0.05) = 6.04$	
	SS	df	QM	QMLF/QMEe = 4.25		$F_4^4(\alpha = 0.05) = 6.39$	
Model	785.61	4	196.40	$r^2 = 0.932$		adjusted $r^2 = 0.898$	
Error	57.54	8	7.19				
Ee	8.52	4	2.13	Optimum A value = 132 rpm			
LF	49.01	4	12.25	Optimum GW = 2.68 g			
Total	843.15	12	70.26	Maximum [AAct]p = 48.60 g/L			

Table S37. Eigenvalues and percentage of explained variance by each component.

Total Variance explained						
	Initial eigenvalues			Extraction Sums of squares loadings		
Component	Eigenvalue	Variance (%)	Cumulative (%)	Total	Variance (%)	Cumulative (%)
1	4.431	40.282	40.28	4.43	40.28	40.28
2	3.563	32.393	72.67	3.56	32.39	72.67
3	1.325	12.041	84.72	1.32	12.04	84.72
4	0.810	7.364	92.08			
5	0.404	3.673	95.75			
6	0.211	1.915	97.67			
7	0.123	1.116	98.78			
8	0.081	0.736	99.52			
9	0.029	0.266	99.79			
10	0.020	0.184	99.97			
11	0.003	0.030	100.00			

Extraction method: principal component analysis.

Table S38. Correlation of beverages with the factors of the PCA based on factor loadings.

Beverages	F1	F2	F3
Bev1-24 h	-1.60	-1.19	-0.44
Bev2-24 h	-1.05	-0.22	0.89
Bev3-24 h	-1.46	0.51	-0.96
Bev4-24 h	-1.03	1.33	-0.19
Bev5-24 h	-1.18	-1.50	1.62
Bev6-24 h	-0.98	0.28	1.44
Bev7-24 h	-1.76	-0.19	-1.92
Bev8-24 h	-1.06	1.04	-0.60
Bev9-24 h	-1.43	-1.00	0.44
Bev1-48 h	0.20	-1.00	-0.67
Bev2-48 h	0.38	-0.17	0.17
Bev3-48 h	0.16	0.94	-0.28
Bev4-48 h	0.22	1.80	0.27
Bev5-48 h	0.31	-1.24	1.13
Bev6-48 h	0.22	1.18	1.73
Bev7-48 h	0.11	0.02	-1.23
Bev8-48 h	0.35	1.39	-0.88
Bev9-48 h	0.24	-0.45	0.85
Bev1-72 h	1.25	-1.51	-1.20
Bev2-72 h	1.13	-0.39	0.45
Bev3-72 h	0.94	0.42	-0.74
Bev4-72 h	0.80	1.33	0.60
Bev5-72 h	1.25	-1.35	0.37
Bev6-72 h	0.86	0.66	0.82
Bev7-72 h	1.09	-0.62	-1.95
Bev8-72 h	1.03	0.61	0.05
Bev9-72 h	1.01	-0.70	0.24

Table S39. Correlation of microbiological and physico-chemical variables with the factors of the PCA based on factor loadings.

**Variables**	**F1**	**F2**	**F3**
TS	-0.663	0.619	0.347
CA	0.272	0.883	-0.187
QA	0.696	0.666	-0.025
LA	-0.763	-0.469	-0.301
AA	0.532	-0.555	-0.466
EtOH	-0.589	-0.021	-0.486
GOH	0.976	-0.046	-0.011
LAB	-0.082	0.668	0.354
AAB	-0.864	-0.347	0.294
Yeasts	0.675	-0.582	0.272
X	0.242	-0.722	0.582
